# Palmitoylation of CLDN12 regulated by ZDHHC7 and APT1/2 promotes hepatitis C virus entry

**DOI:** 10.1128/jvi.00638-26

**Published:** 2026-06-18

**Authors:** Huiying Zhang, Yihan Liu, Ting Xu, Junwen Luan, Leiliang Zhang

**Affiliations:** 1 Department of Clinical Laboratory Medicine, The First Affiliated Hospital of Shandong First Medical University & Shandong Provincial Qianfoshan Hospital 66310, Jinan, Shandong, China; 2 Department of Pathogen Biology, School of Clinical and Basic Medical Sciences, Shandong First Medical University & Shandong Academy of Medical Sciences 518873, Jinan, Shandong, China; 3 Taizhou Center for Disease Control and Prevention, Taizhou, Jiangsu, China; Wake Forest University School of Medicine, Winston-Salem, North Carolina, USA

**Keywords:** hepatitis C virus, CLDN12, palmitoylation, ZDHHC7, virus entry

## Abstract

Hepatitis C virus (HCV) entry is a critical step in establishing infection and is highly dependent on host cell co-receptors. We previously identified that the tight junction protein CLDN12 facilitates HCV entry. However, the regulatory mechanisms governing the dynamic post-translational modifications of CLDN12 remain unclear. This study employs targeted mutagenesis, palmitoylation level analysis, plasma membrane localization, manipulation of key enzymes, and functional virology assays to reveal whether and how reversible palmitoylation regulates the function of CLDN12 in HCV entry. We found that the palmitoylation occurs at cysteine residues C3 and C109 of CLDN12, a modification critical for its role in mediating HCV entry. The palmitoyltransferase ZDHHC7 is a key enzyme that catalyzes the palmitoylation of CLDN12, positively promoting HCV entry. Acyl-protein thioesterases APT1 and APT2 regulate the de-palmitoylation of CLDN12 through differential modulation at the two sites, thereby negatively regulating HCV entry. Our study discovers that ZDHHC7 and APT1/2 form a reversible palmitoylation switch, which regulates the efficiency of HCV entry by dynamically controlling the plasma membrane localization of CLDN12. This finding reveals a new mechanism by which HCV exploits the host’s palmitoylation cycle to promote infection, and highlights this regulatory axis as a potential target for antiviral intervention.

**IMPORTANCE:**

HCV infection poses a significant global health burden. Despite the remarkable efficacy of direct antiviral agents, developing new strategies targeting host factors is crucial for addressing drug resistance and re-infection. Viral entry is a key step in establishing infection, where the host factor CLDN12 promotes HCV invasion. However, the regulatory mechanisms controlling the plasma membrane localization of CLDN12 remain unclear. This study elucidates the mechanism of CLDN12 palmitoylation, regulated by the palmitoyltransferase ZDHHC7 and the acyl-protein thioesterases APT1 and APT2. This dynamic palmitoylation switch governs the plasma membrane localization and function of CLDN12 through reversible modifications, thereby regulating the efficiency of HCV entry. This discovery not only expands our understanding of the network of host palmitoylation regulatory mechanisms involved in HCV entry but also reveals a finely tuned dynamic equilibrium model characterized by the collaborative regulation of palmitoyltransferases and acyl-protein thioesterases, with a preference for specific sites.

## INTRODUCTION

Hepatitis C virus (HCV) infection is one of the major causes of liver cirrhosis and hepatocellular carcinoma (HCC) worldwide, posing a significant threat to human health (1). The advent of direct-acting antiviral agents (DAAs) has profoundly changed the treatment landscape for HCV, achieving a cure rate of over 95% (2). However, achieving global HCV eradication still faces ongoing challenges, including preventing reinfection after cure, addressing rare genotypes, and managing the long-term risk of liver cancer in patients with established cirrhosis (3). Therefore, while maximizing the potential of DAAs, it is essential to actively explore new therapeutic strategies that target different mechanisms of action, particularly those aimed at other stages of the viral life cycle, such as viral entry. Interventions targeting the viral entry phase have shown unique potential (4), not only holding promise for preventing both infection and reinfection but also potentially providing broader antiviral coverage, thus serving as an important complementary strategy for the comprehensive elimination of hepatitis C.

The critical steps for HCV entry into host cells involve the coordinated action of multiple surface receptors: initial attachment is mediated by heparan sulfate proteoglycans (HSPGs) (5, 6) or low-density lipoprotein receptors (LDLR) (7). Subsequently, the HCV envelope protein E2 binds to scavenger receptor class B type I (SR-BI) (8), triggering its cholesterol transferase activity, which promotes the full exposure of E2 epitopes for high-affinity binding to CD81 (9). Next, the virus-receptor complex is transported to the apical membrane domain, maintained by occludin, and mediated by interactions with tight junction proteins (CLDN1/6/9) for clathrin-dependent endocytosis (10, 11). Among these, CD81 is the core receptor for recognition and signaling initiation in the HCV entry process (12). An important host factor, CLDN1, promotes the formation of the CD81-CLDN1 complex via epidermal growth factor receptor (EGFR)-dependent signaling pathways, thus mediating viral entry (13, 14). When CLDN1 is absent, CLDN6 and CLDN9 can serve as alternative co-receptors to support HCV entry into target cells (15–17). Additionally, we previously identified CLDN12 as a novel HCV entry factor that is transported from the endoplasmic reticulum to the Golgi apparatus through a Sec24C-dependent COPII transport pathway, followed by subsequent transport to the plasma membrane (18), similar to CLDN1 (19). However, the mechanisms underlying the plasma membrane targeting of CLDN12 remain unclear.

Palmitoylation is one common mechanism by which proteins localize to the plasma membrane. Mediated by the zinc finger-Asp-His-His-Cys (ZDHHC) family of palmitoyl acyltransferases (PATs) and acyl-protein thioesterases (APTs), palmitoylation can regulate protein localization and plays a crucial role in protein-protein interactions and binding to cholesterol-enriched membrane domains (20). During HCV infection, fatty acid synthase levels are upregulated (21–23), and the palmitic acid produced by fatty acid synthase can provide the substrate for palmitoylation. This critical post-translational modification can not only regulate the life cycle of HCV by modifying the viral protein NS2 (24) but also promote HCV invasion through the palmitoylation of one of its receptors, CD81 (25). However, how palmitoylation regulates CLDN12 and subsequently affects HCV invasion remains largely unexplored.

This study aims to elucidate the mechanism of palmitoylation of the host protein CLDN12 and its role in HCV entry. We have identified the key cysteine modification sites in CLDN12 and analyze how its palmitoylation levels affect its plasma membrane localization and HCV entry efficiency. This study confirmed a comprehensive dynamic regulatory model in which the balance between PATs and APTs functions as a molecular switch. This switch jointly regulates a dynamic and reversible “palmitoylation cycle” that finely tunes the functional state of CLDN12, ultimately influencing the susceptibility of host cells to HCV. Identifying the specific regulatory enzymes involved in this cycle could not only reveal the localization mechanism of CLDN12 but also identify potential antiviral inhibitor targets, thus providing targeted strategies for developing new antiviral entry therapies.

## RESULTS

### Palmitoylation of CLDN12 occurs at cysteine residues 3 and 109

**Fig 1:**
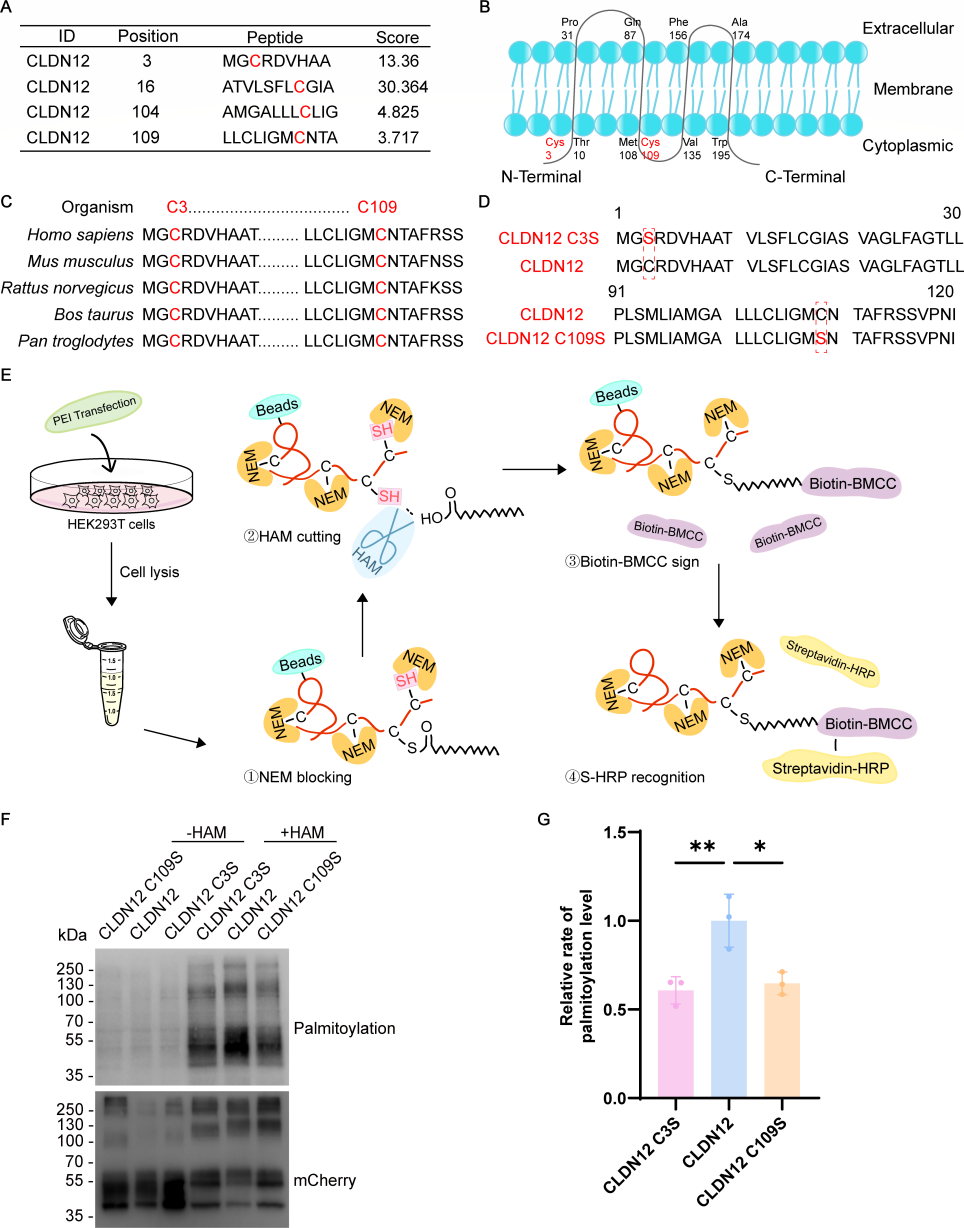
Palmitoylation modifications occur at cysteine residues C3 and C109 of CLDN12. (**A**) Potential palmitoylation sites of the protein CLDN12_HUMAN (UniProtKB: P56749) were predicted using CSS-Palm 4.0. (**B**) Schematic representation of the CLDN12 protein structure, showing that the palmitoylation modification sites (C3, C109) are located on the cytoplasmic side. (**C**) Alignment analysis of CLDN12 amino acid sequences from different species (UniProtKB: human P56749, mouse Q9ET43, rat D4A8Y0, cow Q0IIL2, chimpanzee H2QUW7), with conserved palmitoylation sites marked in red. (**D**) Schematic representation of CLDN12 palmitoylation site mutant construction. Conserved cysteine residues at predicted sites (C3 and C109) were specifically mutated to serine (S). (**E**) Schematic of the acyl-biotin exchange (ABE) experimental workflow. Protein lysates were prepared from HEK-293T cells transfected via PEI and first incubated with buffer containing 10 mM NEM to block free thiols; subsequently treated with hydroxylamine to specifically cleave palmitoyl thioester bonds and expose new thiols; lastly labeled with biotin-BMCC probe and detected by Western blot using streptavidin-HRP. (**F and G**) Detection of palmitoylation levels of wild-type (WT) CLDN12, C3S mutant, and C109S mutant in HEK-293T cells via ABE. Statistical quantification of protein blot results using ImageJ (*n* = 3 independent biological replicates). All data are represented as mean ± standard deviation and normalized to the control group (set to 1). *, *P* < 0.05; **, *P* < 0.01.

The CSS-Palm 4.0 software predicts that the CLDN12 protein contains four cysteine residues likely to undergo palmitoylation at positions 3, 16, 104, and 109 (Fig. 1A). However, we did not select study sites based solely on these prediction scores; instead, we combined them with considerations of substrate accessibility, which is essential for palmitoylation. Palmitoylation is a reversible post-translational modification catalyzed by palmitoyltransferases belonging to the DHHC family. The catalytically active site of these enzymes is located on the cytoplasmic side, meaning that only free sulfhydryl groups (-SH) exposed to the cytoplasm can serve as substrates for this modification. Cysteine residues situated in transmembrane or extracellular regions cannot interact with the enzyme in the cytoplasm, rendering palmitoylation virtually impossible for these residues. As a four-transmembrane protein, the topology of CLDN12 was analyzed using SnapGene software. By inputting its human UniProtKB number (P56749), we obtained a simplified model including its topological domains and transmembrane regions, allowing us to create a structural schematic of CLDN12 (Fig. 1B). This model predicted that the candidate palmitoylation sites C3 and C109 are located within the cytoplasmic domain of the protein, aligning with the biological principle that palmitoylation necessitates access to cytoplasmic palmitoyltransferases. However, it is important to note that this topology is based solely on computational predictions and has not been experimentally validated. According to these predictions, C16 and C104 are likely situated within transmembrane domains. Despite having prediction scores, these residues are embedded in the lipid bilayer, making them inaccessible to cytoplasmic palmitoyltransferases. Thus, true palmitoylation at these sites is virtually impossible under physiological conditions. Consequently, we focused our research on the C3 and C109 sites, which are located on the cytoplasmic side and are accessible to the modifying enzymes (Fig. 1B). We selected these two sites for site-directed mutagenesis and functional validation to investigate the regulatory role of palmitoylation in CLDN12 function. Additionally, we performed multiple sequence alignment analysis to assess the sequence conservation of CLDN12 among different species (Fig. 1C), including human (UniProtKB: P56749), mouse (UniProtKB: Q9ET43), rat (UniProtKB: D4A8Y0), cattle (UniProtKB: Q0IIL2), and chimpanzee (UniProtKB: H2QUW7). The results indicated that both C3 and C109 are highly conserved across the species analyzed. Based on these predictions, we constructed palmitoylation site mutants of CLDN12 (Fig. 1D) by replacing the cysteine residues at the predicted sites (C3 and C109) with serines.

Subsequently, we assessed the palmitoylation level of CLDN12 using the acyl-biotin exchange (ABE) assay (Fig. 1E). Briefly, cell lysates expressing mCherry-tagged CLDN12 were subjected to immunoprecipitation (IP) with agarose beads to enrich the target protein, CLDN12. The IP products were then processed for ABE. The principle of the ABE assay is as follows: free thiols in the protein samples were first blocked with N-ethylmaleimide (NEM). Hydroxylamine (HAM) was then used to cleave the thioester bond between palmitoyl groups and cysteine residues specifically, resulting in the exposure of new free thiols. These newly exposed thiols were subsequently labeled with a biotin-conjugated thiol-reactive probe (Biotin-BMCC). Finally, biotinylated proteins were detected via Western blotting using streptavidin-conjugated horseradish peroxidase (S-HRP). For each sample, a control without HAM (−HAM) was included to account for non-specific labeling. The relative palmitoylation level was quantified as follows: the −HAM control (without thioester cleavage) served as a background control, and the difference or ratio between the +HAM and −HAM signals indicated the palmitoylation level. Additionally, the palmitoylation signal from the +HAM group was normalized to the total protein signal in the corresponding lane (using the same antibody against the target protein). To compare palmitoylation changes under different experimental conditions, the normalized value of each treatment group was further divided by the normalized value of the control group to obtain the relative fold change in palmitoylation.

We overexpressed wild-type mCherry-CLDN12 and its mutants mCherry-CLDN12 C3S and mCherry-CLDN12 C109S in HEK-293T cells and used the ABE assay to assess their palmitoylation levels. The results showed that both mutants exhibited significant reductions in palmitoylation levels compared to the wild type, with decreases of 39.25% (C3S) and 35.34% (C109S) (Fig. 1F and G). This indicates that the cysteine residues C3 and C109 play a critical role in the palmitoylation of CLDN12.

### Palmitoylation of CLDN12 promotes HCV entry

**Fig 2:**
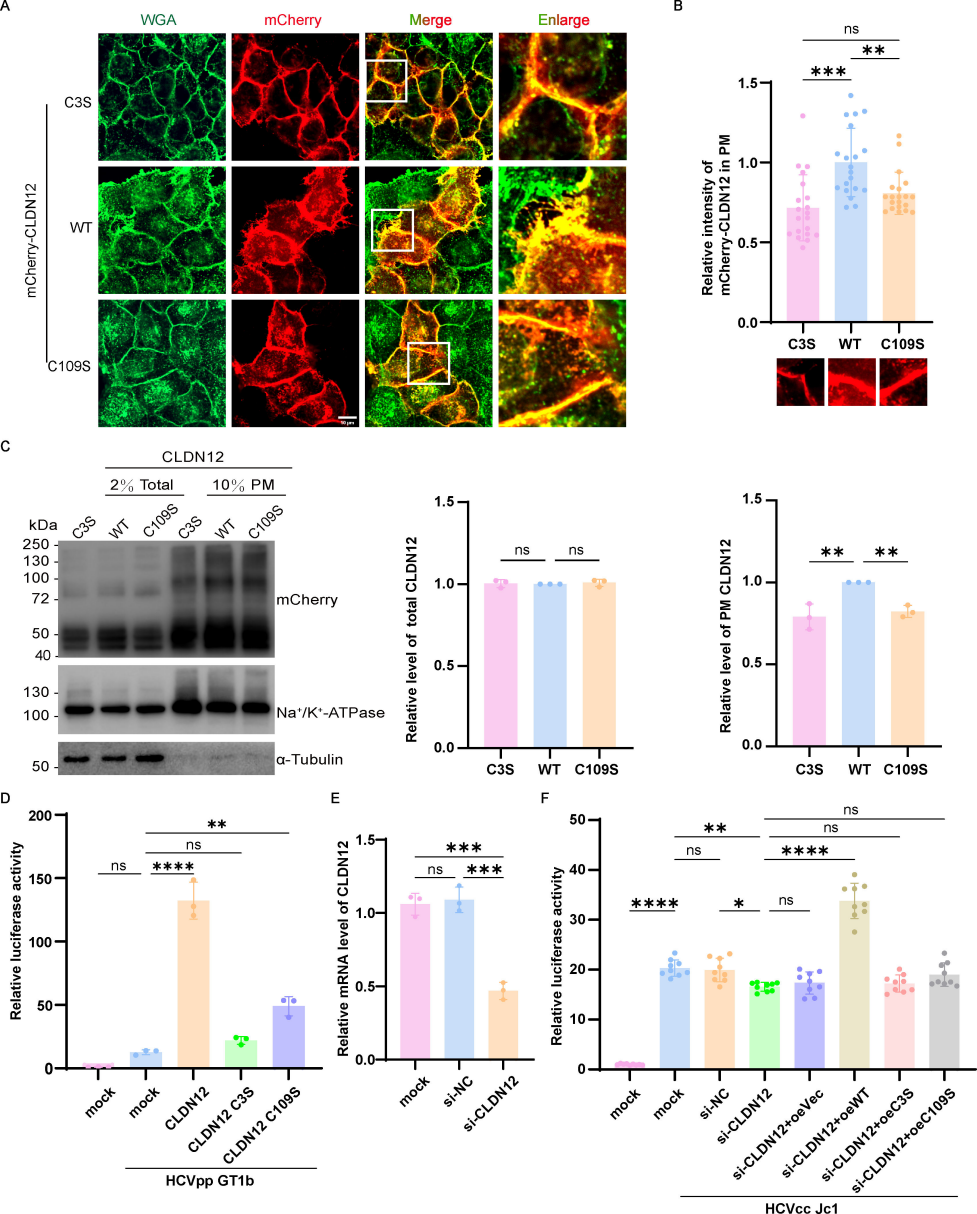
The palmitoylation modification of CLDN12 regulates its role in mediating HCV virus entry. (**A**) Representative immunofluorescence images showing the localization of wild-type (WT) CLDN12 and its palmitoylation site mutants (CLDN12 C3S and CLDN12 C109S) in plasma membrane (PM) marked by wheat germ agglutinin (WGA) in HeLa cells. Scale bar: 10 μm. (**B**) Quantitative analysis of relative mCherry fluorescence intensity in PM using ImageJ (*n* = 20 cells). (**C**) PM enrichment experiment in HeLa cells transfected with CLDN12, CLDN12 C3S, and CLDN12 C109S. Quantitative analysis of relative mCherry blot intensity using ImageJ (*n* = 3 independent biological replicates). (**D**) Effects of CLDN12 palmitoylation site deletion mutants on genotype 1b (GT1b) HCVpp entry efficiency (*n* = 3 independent biological replicates). (**E**) RT-qPCR analysis of CLDN12 mRNA levels in Huh7.5.1 cells transfected with specific siRNA (*n* = 3 independent biological replicates). (**F**) Comparison of HCVcc entry levels under different treatment conditions in Huh7.5.1 cells. Experimental groups included: control, HCVcc-only treatment, si-Control, si-CLDN12, si-CLDN12 + empty vector, si-CLDN12 + OE CLDN12, si-CLDN12 + OE CLDN12 C3S, and si-CLDN12 + OE CLDN12 C109S (*n* = 3 independent biological replicates, with nine technical replicates). All data are presented as mean ± standard deviation and normalized to the control group (set to 1). *, *P* < 0.05; **, *P* < 0.01; ***, *P* < 0.001; ****, *P* < 0.0001; ns indicates no statistical difference.

As a member of the claudins family of tight junction proteins, the level of CLDN12 localization at the plasma membrane influences its efficiency in mediating HCV virus entry. To investigate whether palmitoylation modifies CLDN12’s plasma membrane localization, wild-type mCherry-CLDN12 and the two palmitoylation site mutants (mCherry-CLDN12 C3S and mCherry-CLDN12 C109S) were overexpressed in HeLa cells, followed by immunofluorescence staining and confocal microscopy analysis. The results showed that mCherry-CLDN12 C3S and mCherry-CLDN12 C109S had significantly reduced localization at the plasma membrane, with decreases of 28.40% and 19.28%, respectively, compared to wild-type CLDN12 (Fig. 2A and B). To further validate the role of palmitoylation in the plasma membrane localization of CLDN12, we conducted a plasma membrane enrichment experiment. The results showed that mCherry-CLDN12 C3S and mCherry-CLDN12 C109S exhibited reduced localization at the plasma membrane (Fig. 2C).

To evaluate the effect of palmitoylation on CLDN12-mediated HCV entry, we used an HCV pseudovirus (HCVpp) model for assessment. This model has the advantage of completing only a single infection cycle (the virus entry stage) without replication or generating viral progeny, making it especially suitable for quantitative studies on virus entry. In HEK-293T cells, the infection efficiency of HCVpp was quantified by measuring firefly luciferase activity, while cell viability was assessed using ATP measurement. By normalizing luciferase signals to cell viability, we effectively eliminated experimental bias caused by differences in cell number or potential cytotoxicity, enabling a more accurate reflection of viral entry ability. The experimental results demonstrated that mCherry-CLDN12 C3S and mCherry-CLDN12 C109S exhibited reduced HCVpp entry efficiency by 83.33% and 62.97%, respectively, compared to wild-type CLDN12 (Fig. 2D).

To confirm these findings, we further validated them using the HCV cell culture virus (HCVcc) system. In this experiment, the HCVcc infection efficiency was detected using a *Gaussia* luciferase reporter system, while cell viability was assessed using the CCK-8 method, with normalization of luciferase signals to cell viability to enhance data reliability. To clarify the role of CLDN12 in HCVcc infection, we utilized specific siRNA to knock down endogenous CLDN12 expression in Huh7.5.1 cells, achieving knockdown efficiency of over 57% (Fig. 2E). Subsequently, several experimental conditions were set, including a control group, HCVcc treated alone, si-Control group, si-CLDN12 group, si-CLDN12 + empty vector group, si-CLDN12 + overexpressed wild-type CLDN12 group, si-CLDN12 + overexpressed CLDN12 C3S group, and si-CLDN12 + overexpressed CLDN12 C109S group, to systematically assess the impact of different treatments on HCVcc entry levels. The results showed that, compared to the HCVcc-only group, the si-Control group showed no change in virus entry levels, whereas the si-CLDN12 group showed a reduction of 18.50%. Under conditions of endogenous CLDN12 knockdown, neither the reintroduction of the empty vector, CLDN12 C3S, nor CLDN12 C109S significantly restored the viral entry efficiency, whereas reintroducing wild-type CLDN12 significantly enhanced the viral entry levels (Fig. 2F). Taken together, these findings suggest that palmitoylation modification of CLDN12 plays a crucial regulatory role in its mediation of HCV virus entry into host cells.

### ZDHHC7 is the key PAT regulating CLDN12 palmitoylation

**Fig 3:**
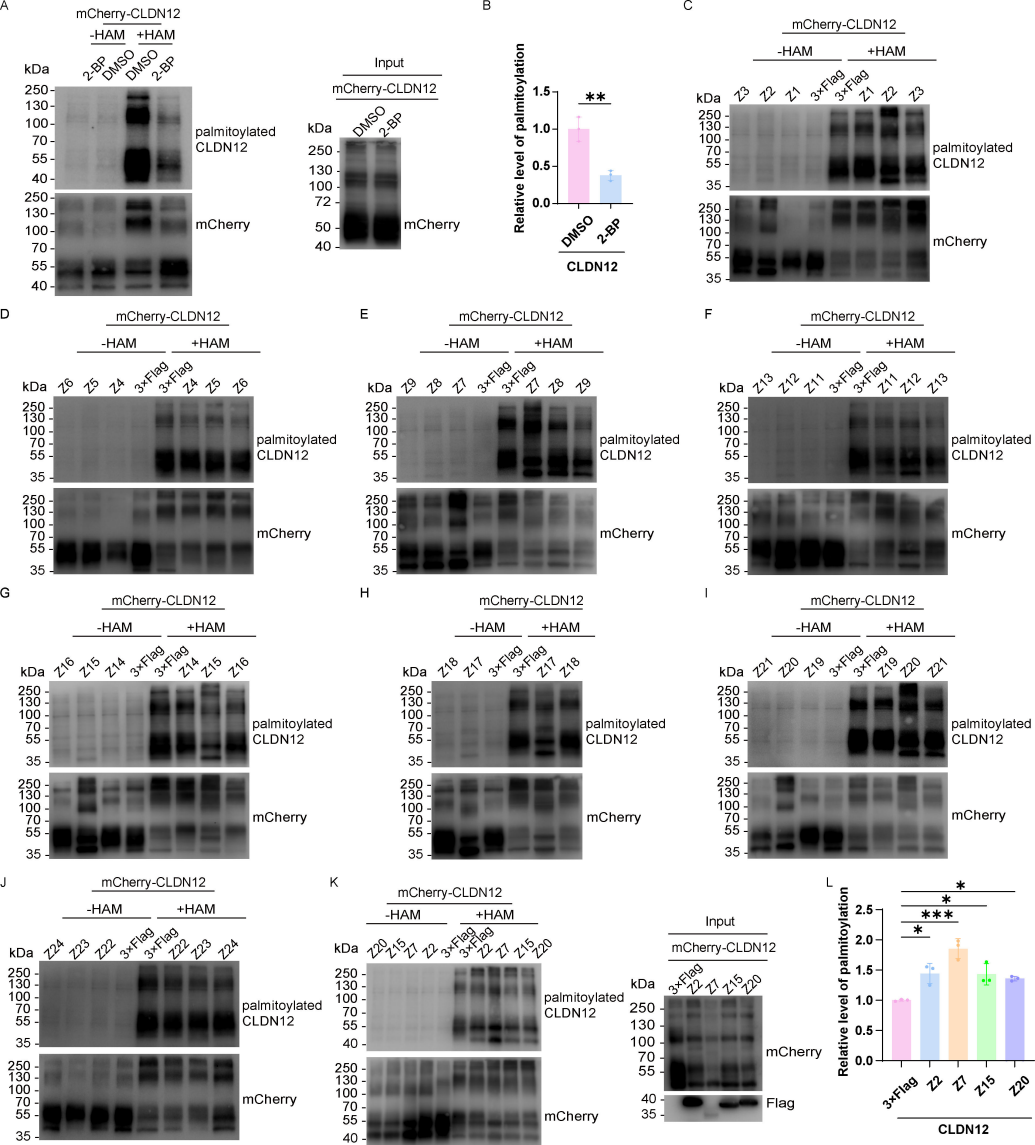
Screening of palmitoyl transferases regulating CLDN12 palmitoylation. (**A and B**) In HEK-293T cells transfected with WT CLDN12, changes in CLDN12 palmitoylation levels were detected through ABE experiments following treatment with the broad-spectrum palmitoylation inhibitor 2-BP (50 μM) or solvent DMSO. (**B**) Statistical analysis of Western blot results using ImageJ (*n* = 3 independent biological replicates). (**C–J**) mCherry-tagged CLDN12 was co-transfected with Flag-tagged ZDHHC enzymes (23 types) into HEK-293T cells. After cell lysis, ABE experiments were performed to detect CLDN12 palmitoylation levels. (**K–L**) The effects of four initially screened palmitoyl transferases (ZDHHC2, ZDHHC7, ZDHHC15, ZDHHC20) on CLDN12 palmitoylation levels were examined via ABE experiments, revealing that ZDHHC7 had the most significant regulatory effect. (**D**) Statistical analysis of protein blot results using ImageJ (*n* = 3 independent biological replicates). All data are presented as mean ± standard deviation and normalized to the control group (set to 1). *, *P* < 0.05; **, *P* < 0.01; ***, *P* < 0.001; ****, *P* < 0.0001; ns indicates no statistical difference.

PATs are key enzymes that catalyze the palmitoylation of proteins. 2-Bromopalmitate (2-BP), a broad-spectrum palmitoyltransferase inhibitor, bears a chemical structure highly similar to natural palmitate, allowing it to competitively bind to the catalytic center of PATs and thus block the binding of palmitoyl-CoA to the enzyme, reversibly inhibiting the transfer of palmitoyl groups to target proteins (26, 27). To explore the enzymatic mechanism of CLDN12 palmitoylation, we overexpressed mCherry-CLDN12 in HEK-293T cells and treated the cells with 2-BP. The results indicated that 2-BP treatment reduced the palmitoylation levels of CLDN12 by 62.12%, confirming that this modification depends on the catalytic activity of PATs (Fig. 3A and B). Next, we screened which palmitoyltransferases participated in the palmitoylation of CLDN12 using the ABE experimental system. Among 23 palmitoyltransferases, we identified four candidate enzymes potentially involved in the palmitoylation modification of CLDN12: ZDHHC2, ZDHHC7, ZDHHC15, and ZDHHC20 (Fig. 3C–3J). To validate the regulatory effects of these enzymes on CLDN12 palmitoylation levels, we further assessed their impact on the extent of CLDN12 palmitoylation. The results demonstrated that all four ZDHHC family members could increase the palmitoylation levels of CLDN12 to varying degrees, with ZDHHC7 exerting the most significant regulatory effect (Fig. 3K and L).

**Fig 4:**
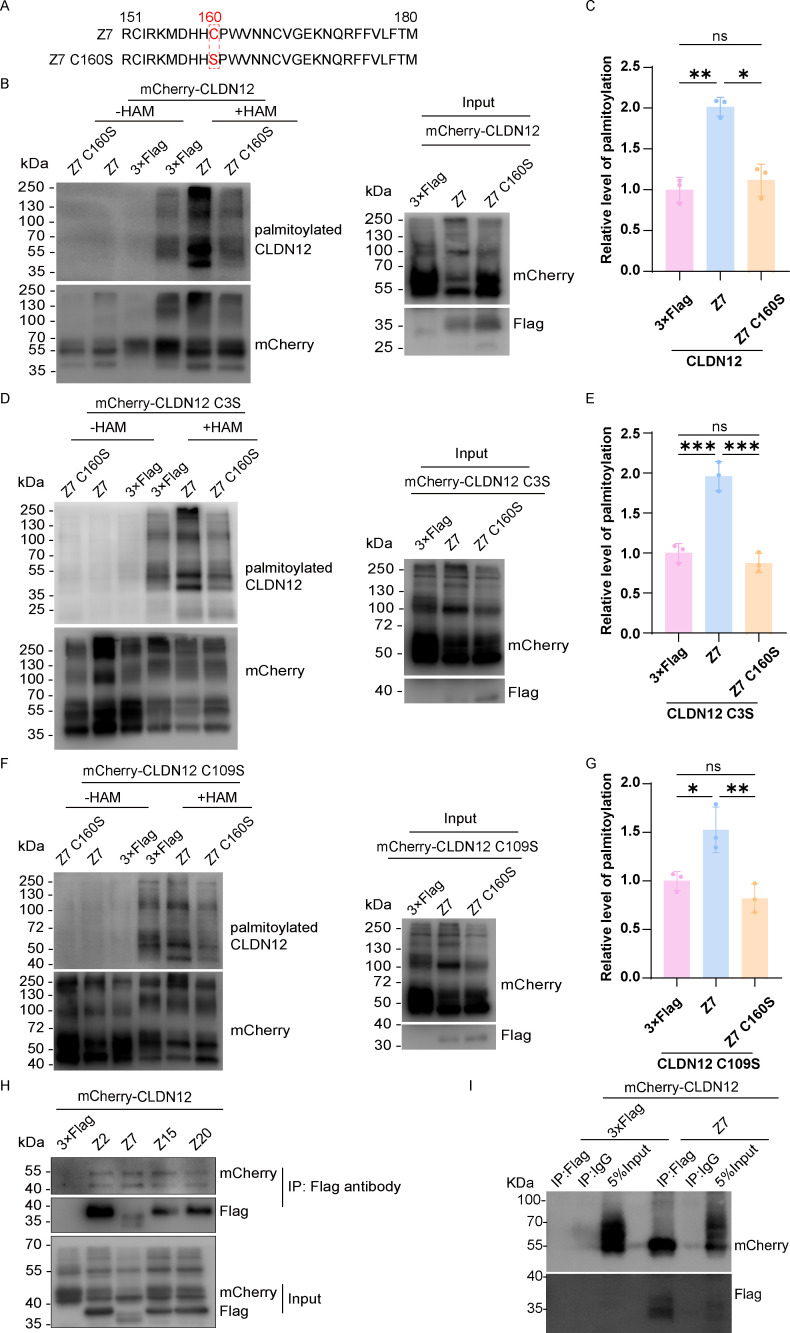
ZDHHC7 regulates CLDN12 palmitoylation. (**A**) Schematic representation of ZDHHC7 enzyme activity mutant construction (C160S) (UniProtKB: Q9NXF8). This mutation replaces the conserved cysteine residue (C160, red) in the predicted S-palmitoyl intermediate site with serine. (**B–G**) The effects of overexpressing WT ZDHHC7 and its enzyme activity mutant ZDHHC7 C160S on palmitoylation levels of WT CLDN12 and its palmitoylation site mutants (C3S, C109S) were assessed in HEK-293T cells. Statistical analysis of protein blot results using ImageJ (*n* = 3 independent biological replicates) (**C, E, G**). (**H**) Co-immunoprecipitation experiments confirmed the protein interaction between ZDHHCs and CLDN12. (**I**) Co-immunoprecipitation experiments confirmed the protein interaction between ZDHHC7 and CLDN12. All data are presented as mean ± standard deviation and normalized to the control group (set to 1). *, *P* < 0.05; **, *P* < 0.01; ***, *P* < 0.001; ns indicates no statistical difference.

Using the human UniProtKB number of ZDHHC7 (Q9NXF8), we generated a simplified structural map with SnapGene software. The analysis revealed that cysteine residue 160 located within the DHHC zinc finger domain, which is in the enzyme’s catalytic center, is a critical site for forming the S-palmitoylation intermediate and plays a decisive role in ZDHHC7’s palmitoyltransferase activity. We employed site-directed mutagenesis to replace this conserved cysteine with serine, successfully generating a ZDHHC7 catalytic inactive mutant (Fig. 4A). To systematically evaluate the impact of ZDHHC7 and its catalytically inactive mutant (C160S) on the palmitoylation of wild-type CLDN12 and the two palmitoylation site mutants, we conducted ABE experiments in HEK-293T cells. The results indicated that overexpression of wild-type ZDHHC7 significantly enhanced the palmitoylation levels of CLDN12 and its two mutants (Fig. 4B through G). In contrast, mutation of the key catalytic residue (C160) within the DHHC domain of ZDHHC7 completely abolished its capacity to modify palmitoylation in CLDN12 and the mutants (Fig. 4B through G). These findings suggest that ZDHHC7 participates in the regulation of CLDN12 palmitoylation level through its unique catalytic activity. To confirm the interaction between the four initially screened ZDHHC proteins and CLDN12, we co-transfected Flag-tagged ZDHHC proteins with mCherry-tagged CLDN12 in HEK-293T cells. After 24 h of transfection, cell lysates were collected for co-immunoprecipitation experiments. As shown in Fig. 4H and I, the immunoprecipitation results confirm that ZDHHC2, ZDHHC7, ZDHHC15, and ZDHHC20 are all capable of interacting with CLDN12.

### ZDHHC7-dependent palmitoylation of CLDN12 promotes HCV entry

**Fig 5:**
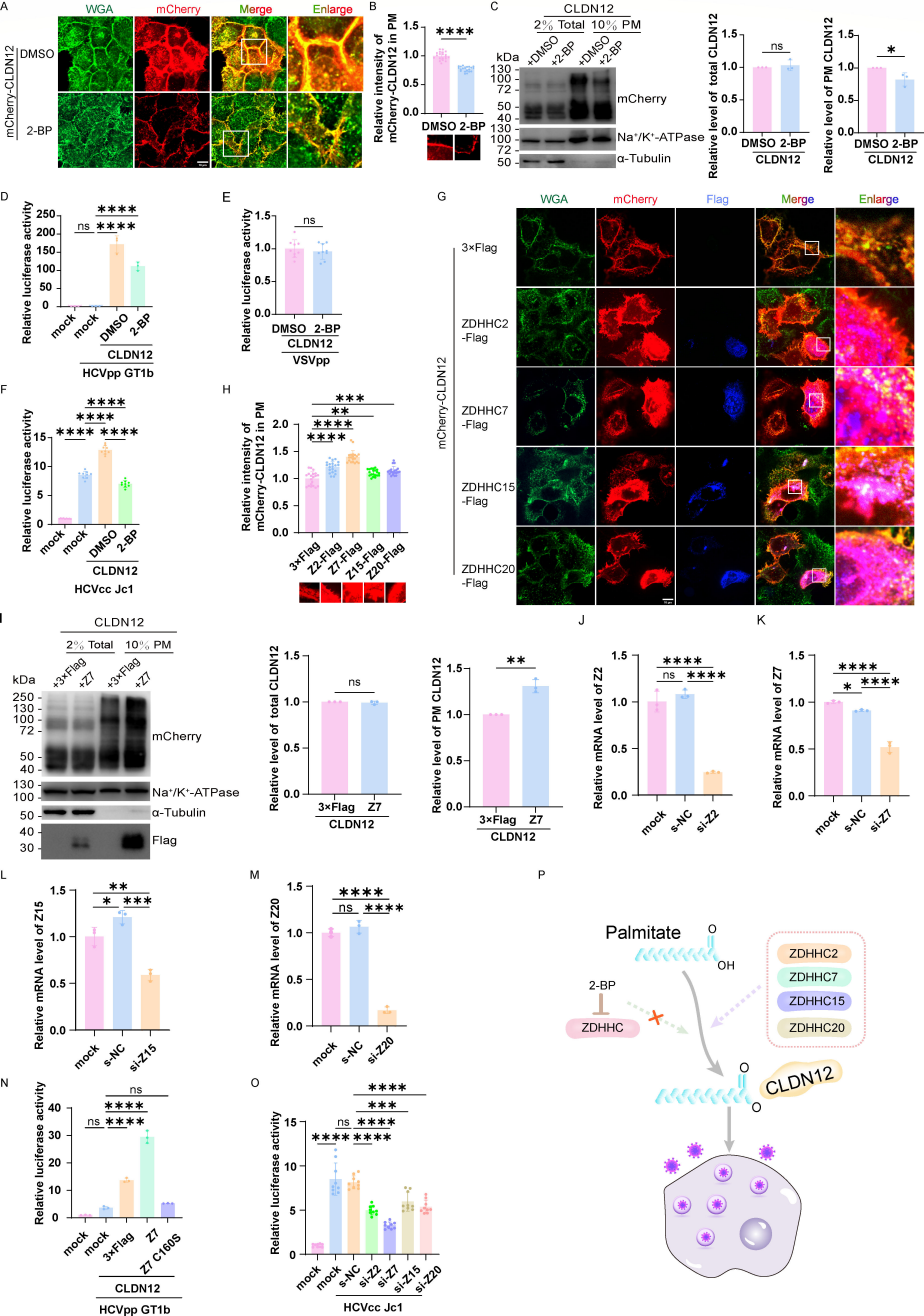
ZDHHC7 regulates CLDN12-mediated HCV virus entry. (**A**) Representative images showing co-localization of WT CLDN12 in HeLa cells treated with 2-BP (50 μM) or DMSO, with CLDN12 (red) and WGA (green). Scale bar: 10 μm. (**B**) Quantitative analysis of relative intensity of mCherry fluorescence signal in PM from panel **A** (*n* = 20 cells). (**C**) PM enrichment experiment in HeLa cells transfected with CLDN12 in the presence of 2-BP or DMSO. Quantitative analysis of relative mCherry blots intensity using ImageJ (*n* = 3 independent biological replicates). (**D**) Effects of 2-BP and DMSO treatment on HCVpp entry efficiency mediated by CLDN12 in HEK-293T cells (*n* = 3 independent biological replicates). (**E**) Effects of 2-BP and DMSO treatment on VSVpp entry efficiency mediated by CLDN12 in HEK-293T cells (*n* = 8 independent biological replicates). (**F**) Assessment of the effects of 2-BP treatment on HCVcc entry efficiency in Huh7.5.1 cells using HCVcc infection model. Experimental groups included the following: control, HCVcc-only treatment, DMSO, and 2-BP (*n* = 3 independent biological replicates, with nine technical replicates). (**G**) Co-localization analysis of co-transfected CLDN12 (red) with empty vector (3 × Flag) and four palmitoyl transferases (ZDHHC2, 7, 15, 20; blue) in HeLa cells and WGA (green). Scale bar: 10 μm. (**H**) Quantitative analysis of relative intensity of mCherry fluorescence signal in PM from panel G (*n* = 20 cells). (**I**) PM enrichment experiment in HeLa cells transfected with CLDN12/3 × Flag or CLDN12/ZDHHC7. Quantitative analysis of relative mCherry blots intensity using ImageJ (*n* = 3 independent biological replicates). (**J–M**) RT-qPCR analysis of mRNA expression levels of ZDHHC2 (**J**), ZDHHC7 (**K**), ZDHHC15 (**L**), and ZDHHC20 (**M**), following specific siRNA knockdown in Huh7.5.1 cells (*n* = 3 independent biological replicates). (**N**) The effects of co-transfection of CLDN12 with empty vector (3 × Flag), WT ZDHHC7 (ZDHHC7-Flag), or its enzyme activity mutant (ZDHHC7 C160S-Flag) on CLDN12-mediated HCVpp entry efficiency were examined in HEK-293T cells (*n* = 3 independent biological replicates). (**O**) Evaluation of the effects of knockdown of different PATs on HCVcc entry efficiency in Huh7.5.1 cells. Experimental groups included the following: control, HCVcc-only treatment, si-Control, si-ZDHHC2, si-ZDHHC7, si-ZDHHC15, and si-ZDHHC20 (*n* = 3 independent biological replicates, with nine technical replicates). (**P**) PATs (represented by ZDHHC7) promote HCV entry by increasing CLDN12 palmitoylation levels, while 2-BP blocks this process by inhibiting PAT enzymatic activity. All data are presented as mean ± standard deviation and normalized to the control group (set to 1). *, *P* < 0.05; **, *P* < 0.01; ***, *P* < 0.001; ****, *P* < 0.0001; ns indicates no statistical difference.

To investigate the impact of 2-BP on CLDN12 subcellular localization, we overexpressed wild-type mCherry-CLDN12 in HeLa cells and treated them with 2-BP, followed by analysis through immunofluorescence staining and confocal microscopy. The results showed that after 2-BP treatment, the level of CLDN12 at the plasma membrane was reduced by 22.80% (Fig. 5A and B), indicating that the stable anchoring of CLDN12 at the plasma membrane relies on the active maintenance mediated by PAT enzyme-catalyzed palmitoylation. Plasma membrane enrichment experiments further confirmed that 2-BP decreased the plasma membrane localization of CLDN12 (Fig. 5C). Therefore, the catalytic activity of PAT enzymes is indispensable in regulating CLDN12’s subcellular distribution.

Based on these findings, we further explored the effect of 2-BP on the biological function of CLDN12, aiming to elucidate its molecular mechanism, wherein competitive inhibition of PAT enzymes modulates palmitoylation to influence the efficiency of CLDN12-mediated virus entry. Using the four-plasmid system to package HCVpp, we infected CLDN12 overexpressing cells treated with 2-BP as well as untreated cells. The results demonstrated that 2-BP treatment reduced the entry efficiency of HCVpp by 35.17%, indicating that it inhibits virus entry by weakening CLDN12 palmitoylation levels and its plasma membrane localization (Fig. 5D). There was no significant change in VSVpp entry efficiency, further indicating that CLDN12-mediated palmitoylation specifically regulates HCV entry (Fig. 5E). Further validation in the HCVcc model, which closely simulates physiological infection conditions, consistently confirmed that 2-BP significantly weakened CLDN12-mediated HCV virus entry by 45.68% (Fig. 5F).

Given that 2-BP, as a broad-spectrum inhibitor, lacks specificity, we aimed to identify the specific PAT enzymes regulating CLDN12 plasma membrane localization. We overexpressed four candidate PAT enzymes (ZDHHC2, ZDHHC7, ZDHHC15, and ZDHHC20) along with wild-type CLDN12 in HeLa cells and systematically analyzed their effects using immunofluorescence staining and confocal microscopy. The results showed that all four PAT enzymes could promote the localization of CLDN12 at the plasma membrane to varying degrees, with ZDHHC7 exhibiting the most significant enhancement (Fig. 5G and H). Plasma membrane enrichment experiments further confirmed that ZDHHC7 increased the plasma membrane localization of CLDN12 (Fig. 5I). This phenomenon correlates closely with the previous ABE experiments indicating that ZDHHC7 has the strongest enhancing effect on CLDN12 palmitoylation, thus further confirming the core role of ZDHHC7 in regulating CLDN12 palmitoylation and function at the protein subcellular localization level.

To further validate the effects of the four PAT enzymes on CLDN12-mediated virus infection efficiency in a physiological context using the HCVcc model, we employed specific siRNA to knock down endogenous expression of ZDHHC2, ZDHHC7, ZDHHC15, and ZDHHC20 in Huh7.5.1 cells. The experiments confirmed the knockdown efficiencies for each gene: Z2 at 77.36%, Z7 at 42.98%, Z15 at 51.24%, and Z20 at 84.16% (Fig. 5J–M).

Next, to explore the effects of ZDHHC7 and its catalytic activity on CLDN12-mediated virus entry, we infected HEK-293T cells overexpressing CLDN12 together with an empty vector, wild-type ZDHHC7, or its catalytically inactive mutant, with HCVpp. The experimental results indicated that wild-type ZDHHC7 significantly enhanced the HCVpp entry efficiency mediated by CLDN12, while its enzymatic activity mutant did not produce a significant effect on CLDN12-mediated virus entry (Fig. 5N).

To verify the functional roles of specific PAT enzymes in HCV infection, we infected Huh7.5.1 cells with HCVcc after knocking down the four PAT enzymes and detected the virus infection efficiency using a *Gaussia* luciferase reporter system. The results showed that knocking down PAT enzyme expression significantly inhibited HCVcc infection, with the regulatory effect of ZDHHC7 being the most pronounced (Fig. 5O). This finding further indicates that the catalytic activity of PAT enzymes plays a crucial role in CLDN12-mediated virus entry, thereby functionally establishing ZDHHC7 as a key factor regulating CLDN12-mediated HCV virus entry.

Figure 5P presents the molecular mechanism model illustrating how palmitoyltransferases influence HCV entry by regulating CLDN12 palmitoylation levels. Specifically, the PAT enzyme (represented by ZDHHC7) catalyzes CLDN12 palmitoylation, thereby promoting HCV virus entry. Conversely, the palmitoylation inhibitor 2-BP effectively blocks this process by inhibiting PAT enzyme catalytic activity.

### APT1 and APT2 are APTs regulating CLDN12 palmitoylation and exhibit differential regulation of C3 and C109 sites

**Fig 6:**
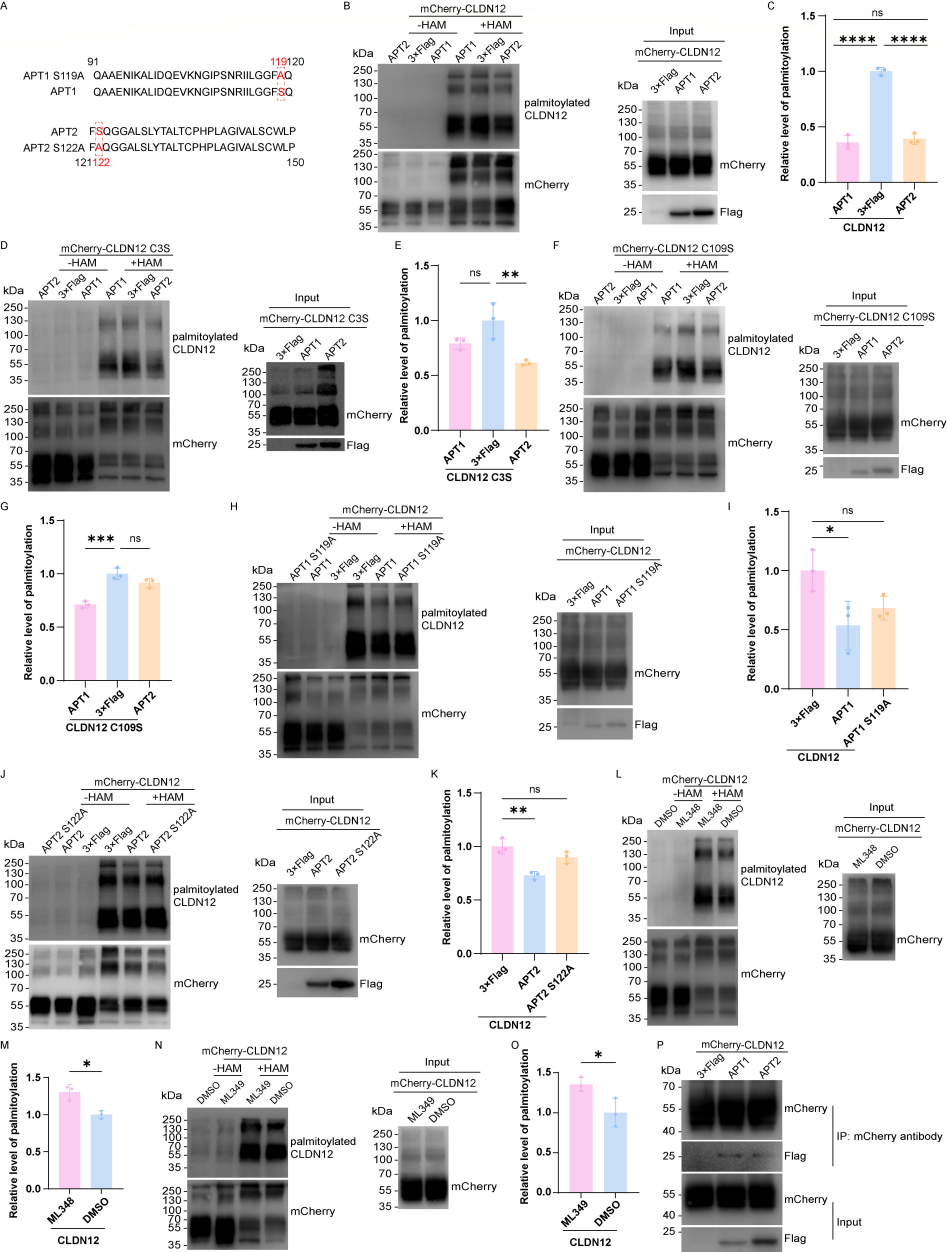
APT1 and APT2 regulate CLDN12 palmitoylation with differential effects on C3 and C109 sites. (**A**) Schematic representation of the active site mutants (S119A/S122A) of APT1 and APT2 thioesterase (UniProtKB: APT1 O75608; APT2 O95372). Key serine residues (S119/S122, red) in the catalytic center were mutated to alanine (A). (**B–G**) Overexpression of WT APT1 or APT2 in HEK-293T cells followed by ABE experiments to detect their effects on WT CLDN12 (**B**), CLDN12 C3S (**D**), and CLDN12 C109S (**F**) palmitoylation levels. Statistical analysis of protein blot results using ImageJ (**C, E, G**) (*n* = 3 independent biological replicates). (**H–K**) Overexpression of WT APT1 (**H**) or APT2 (**J**) and their corresponding enzyme activity mutants in HEK-293T cells to examine their effects on WT CLDN12 palmitoylation levels. (**I, K**) Statistical analysis of protein blot results using ImageJ (**I, K**) (*n* = 3 independent biological replicates). (**L–O**) In HEK-293T cells expressing CLDN12, treatment with APT1 inhibitor ML348 (5 μM, **L**) or APT2 inhibitor ML349 (5 μM, **N**) was performed to analyze CLDN12 palmitoylation levels via ABE experiments. Statistical analysis of protein blot results using ImageJ (**M, O**) (*n* = 3 independent biological replicates). (**P**) Co-immunoprecipitation experiments verifying interactions between APT1/APT2 and CLDN12. Data are obtained from three independent biological replicates and expressed as mean ± standard deviation, normalized to the control group (set to 1). *t*-tests were used for comparisons between two groups, and one-way ANOVA was used for comparisons among three or more groups. *, *P* < 0.05; **, *P* < 0.01; ***, *P* < 0.001; ****, *P* < 0.0001; ns indicates no statistical difference.

APTs are key enzymes that achieve precise depalmitoylation of proteins. They dynamically regulate protein function and subcellular localization by hydrolyzing the thioester bond linking fatty acyl chains (like palmitoyl) to cysteine residues in proteins. Based on the sequences of human acyl protein thioesterases APT1 (UniProtKB: Q9NXF8) and APT2 (UniProtKB: O95372), we generated simplified structural maps for both using SnapGene software, identifying S119 and S122 as the thioesterase active sites for each. We constructed the corresponding inactive mutants by site-directed mutagenesis, replacing the serine residues at these sites with alanine (A) (Fig. 6A). To explore the effects of APT1 and APT2 on CLDN12, we co-transfected plasmids expressing mCherry-CLDN12 with APT1-Flag or APT2-Flag in HEK-293T cells and assessed CLDN12 palmitoylation levels using the ABE assay. The results indicated that both thioesterases effectively depalmitoylated CLDN12, with APT1 reducing palmitoylation by 64.03% and APT2 by 60.89% (Fig. 6B and C). To investigate whether APT1 and APT2 regulate different palmitoylation sites on CLDN12, we further overexpressed the two single palmitoylation site mutants (C3S and C109S) in HEK-293T cells after co-transfection with APT1 or APT2 and conducted ABE experiments. The results indicated that APT1 primarily regulates the palmitoylation of the C3 site, while APT2 mainly acts on the C109 site, suggesting that they exert differential regulation on the palmitoylation sites of CLDN12 (Fig. 6D through G).

To investigate the effects of APTs and their thioesterase active site mutants (S119A, S122A) on wild-type CLDN12 palmitoylation, we performed ABE experiments in HEK-293T cells. The results showed that overexpression of wild-type APT1 and APT2 significantly reduced the palmitoylation levels of CLDN12 (Fig. 6H through K). Interestingly, both the APT1-S119A and APT2-S122A mutants also exhibited reduced palmitoylation of CLDN12, although to a lesser degree compared to their wild-type versions. This suggests that both mutants still possess some residual activity (Fig. 6I and K). These results indicate that the regulation of CLDN12 by APTs is primarily driven by a direct enzymatic reaction that relies on their thioesterase activity.

ML348 and ML349 are small-molecule inhibitors that specifically target acyl protein thioesterases APT1 and APT2, respectively (28). They achieve reverse regulation of protein palmitoylation levels by specifically inhibiting the activity of depalmitoylating enzymes. After overexpressing CLDN12 in HEK-293T cells and treating them with either inhibitor, subsequent ABE experiments revealed that CLDN12 palmitoylation levels significantly increased after treatment with both inhibitors (Fig. 6L through O), corroborating the results that overexpression of APT1 and APT2 reduces CLDN12 palmitoylation levels and reinforcing the notion that cells utilize the cooperative regulatory action of these two enzymes to maintain a dynamic balance of CLDN12 palmitoylation.

To confirm the interaction between APT1/2 and CLDN12, we transfected HEK-293T cells with plasmids expressing Flag-tagged APT1 or APT2, along with mCherry-tagged CLDN12. After 24 h of transfection, the cell lysates were collected for immunoprecipitation experiments. The results shown in Fig. 6P indicate that both APT1 and APT2 can specifically bind to CLDN12, confirming a direct interaction between APT1, APT2, and CLDN12 in cells, and suggesting that APT1 and APT2 may precisely influence CLDN12 as a cell surface receptor by regulating its dynamic palmitoylation.

### APT1 and APT2 negatively regulate CLDN12-mediated HCV entry

**Fig 7:**
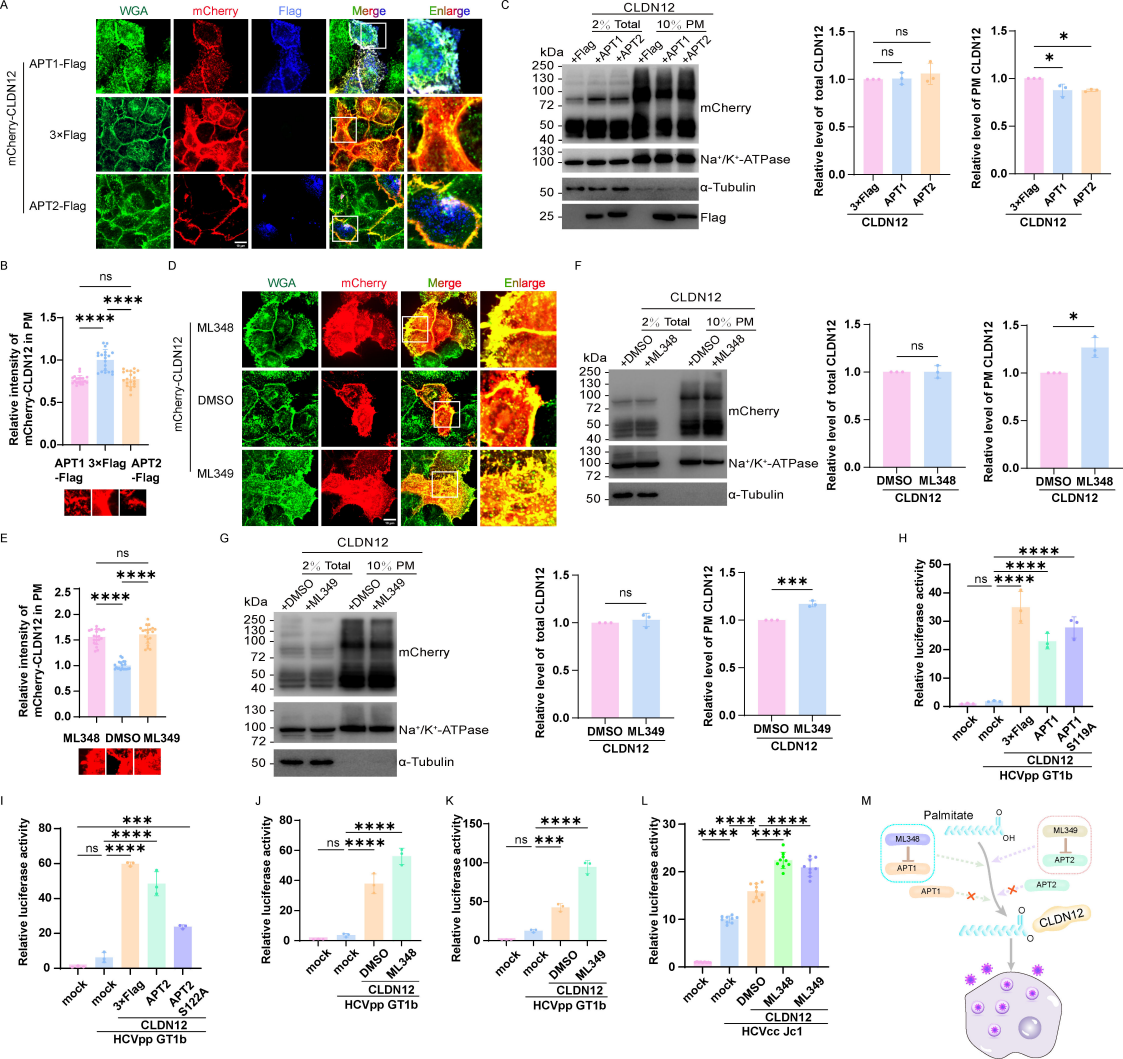
APT1 and APT2 regulate CLDN12-mediated HCV entry. (**A and B**) Localization analysis of CLDN12 (red) in PM (WGA, green) in the presence of empty vector (3 × Flag), APT1, or APT2 (blue) in HeLa cells transfected simultaneously. Scale bar: 10 μm. (**B**) Quantitative analysis of relative intensity of mCherry fluorescence signal in PM from panel A (*n* = 20 cells). (**C**) PM enrichment experiment in HeLa cells transfected with CLDN12/3 × Flag, or CLDN12/APT1, or CLDN12/APT2. Quantitative analysis of relative mCherry blots intensity using ImageJ (*n* = 3 independent biological replicates). (**D**) CLDN12-expressing HeLa cells were treated with ML348, ML349, or DMSO followed by the localization analysis of CLDN12 (red) in PM marked by WGA (green). Scale bar: 10 μm. (**E**) Quantitative analysis of relative intensity of mCherry fluorescence signal in PM from panel D (*n* = 20 cells). (**F**) PM enrichment experiment in HeLa cells transfected with CLDN12 in the presence of ML348 or DMSO. Quantitative analysis of relative mCherry blots intensity using ImageJ (*n* = 3 independent biological replicates). (**G**) PM enrichment experiment in HeLa cells transfected with CLDN12 in the presence of ML349 or DMSO. Quantitative analysis of relative mCherry blots intensity using ImageJ (*n* = 3 independent biological replicates). (**H and I**) Overexpression of WT and enzyme activity mutants of APT1 (**H**) or APT2 (**I**) in HEK-293T cells to assess their effects on CLDN12-mediated HCVpp entry efficiency (*n* = 3 independent biological replicates). (**J and K**) In CLDN12-expressing HEK-293T cells, treatment with APT1 inhibitor ML348 (**J**) or APT2 inhibitor ML349 (**K**) was used to evaluate their effects on CLDN12-mediated HCVpp entry efficiency (*n* = 3 independent biological replicates). (**L**) Assessment of the effects of ML348 and ML349 treatments on HCVcc entry efficiency in Huh7.5.1 cells within an HCVcc infection model. Experimental groups included the following: control, HCVcc-only treatment, DMSO, ML348, and ML349 (*n* = 3 independent biological replicates, with nine technical replicates). (**M**) APT1/APT2 negatively regulate HCV entry by mediating CLDN12 de-palmitoylation. ML348 and ML349 block this process by inhibiting the enzymatic functions of APT1/APT2. All data are derived from three independent biological replicates and are presented as mean ± standard deviation, normalized relative to the control group (set to 1). Comparisons among multiple groups were performed using one-way ANOVA. ***, *P* < 0.001; ****, *P* < 0.0001; ns indicates no statistical difference.

To further investigate the regulatory roles of APT1 and APT2 in CLDN12 subcellular localization, we overexpressed plasmids expressing mCherry-tagged CLDN12 along with Flag-tagged APT1 or APT2 in HeLa cells and analyzed them using immunofluorescence staining and confocal microscopy. The results indicated that both APT1 and APT2 significantly reduced the localization of CLDN12 at the plasma membrane, with APT1 decreasing localization by 23.24% and APT2 by 22.34% (Fig. 7A and B). Plasma membrane enrichment experiments further demonstrated that both APT1 and APT2 decreased the localization of CLDN12 at the plasma membrane (Fig. 7C). Therefore, the stable presence of CLDN12 at the plasma membrane depends on the dynamic and reversible palmitoylation modifications. APT1 and APT2, as depalmitoylating enzymes, achieve precise negative regulation of CLDN12 membrane localization through modulation of this modification process. To further validate this regulatory mechanism, we applied acyl protein thioesterase inhibitors ML348 and ML349 to HeLa cells overexpressing CLDN12 and observed similar changes in localization. The results showed that treatment with both inhibitors significantly enhanced CLDN12’s localization at the plasma membrane (Fig. 7D through G), thereby confirming the important roles of APT1 and APT2 in regulating CLDN12’s palmitoylation level and function.

To explore the mechanisms by which APT1 and APT2 influence CLDN12’s plasma membrane localization and HCV receptor function via modulation of its palmitoylation levels, we utilized the HCVpp model to infect cells overexpressing wild-type CLDN12, APT1 and its enzymatic activity mutant (S119A), and APT2 and its enzymatic activity mutant (S122A), and analyzed them using a luciferase reporter system (normalized to cellular ATP levels). The results indicated that both APT1 and APT2 significantly inhibited CLDN12-mediated HCVpp entry, with APT1 causing a reduction of 34.19% and APT2 a reduction of 18.92% (Fig. 7H). Notably, APT1 enzyme activity mutant could restore HCVpp entry function (Fig. 7H), indicating that its negative regulation of CLDN12 is completely dependent on depalmitoylation activity, consistent with its biochemical profile. However, the APT2 enzyme activity mutant did not exhibit a compensatory effect (Fig. 7I), suggesting a more complex functional mechanism. This mutant may retain binding ability to CLDN12 but lack catalytic function, forming stable “inactive complexes,” resulting in a deleterious negative effect: not only is its own function become impaired, but it may also competitively interfere with endogenous APT2 or other regulatory factors’ normal handling of CLDN12, leading to a more pronounced suppression of HCVpp entry. Additionally, in CLDN12-overexpressing cells, compared to the dimethylsulfoxide (DMSO) treatment group, the HCVpp entry efficiency was significantly increased in the ML348 or ML349 treatment groups (Fig. 7J and K), indicating that inhibiting depalmitoylating enzyme activity promotes CLDN12 palmitoylation, facilitating HCV entry. In summary, APT1 and APT2 regulate CLDN12 palmitoylation through their depalmitoylating enzyme activity, influencing HCV entry.

We subsequently infected Huh7.5.1 cells treated with ML348 and ML349 with HCVcc and assessed virus infection efficiency using a *Gaussia* luciferase reporter system. The results indicated that, after normalizing to cell viability, treatment with both inhibitors significantly enhanced HCVcc infection levels (Fig. 7L). This result once again functionally confirms that APT1 and APT2 are key factors negatively regulating HCV virus entry through their depalmitoylating activities on CLDN12. Fig. 7M illustrates the molecular mechanism model, whereby acyl protein thioesterases and their inhibitors regulate CLDN12 palmitoylation modifications, consequently influencing HCV entry. Specifically, APT enzymes negatively regulate HCV entry by depalmitoylating CLDN12. Their inhibitors, on the other hand, relieve the suppression of CLDN12 function by blocking APT enzyme activity, thus promoting virus entry.

## DISCUSSION

**Fig 8:**
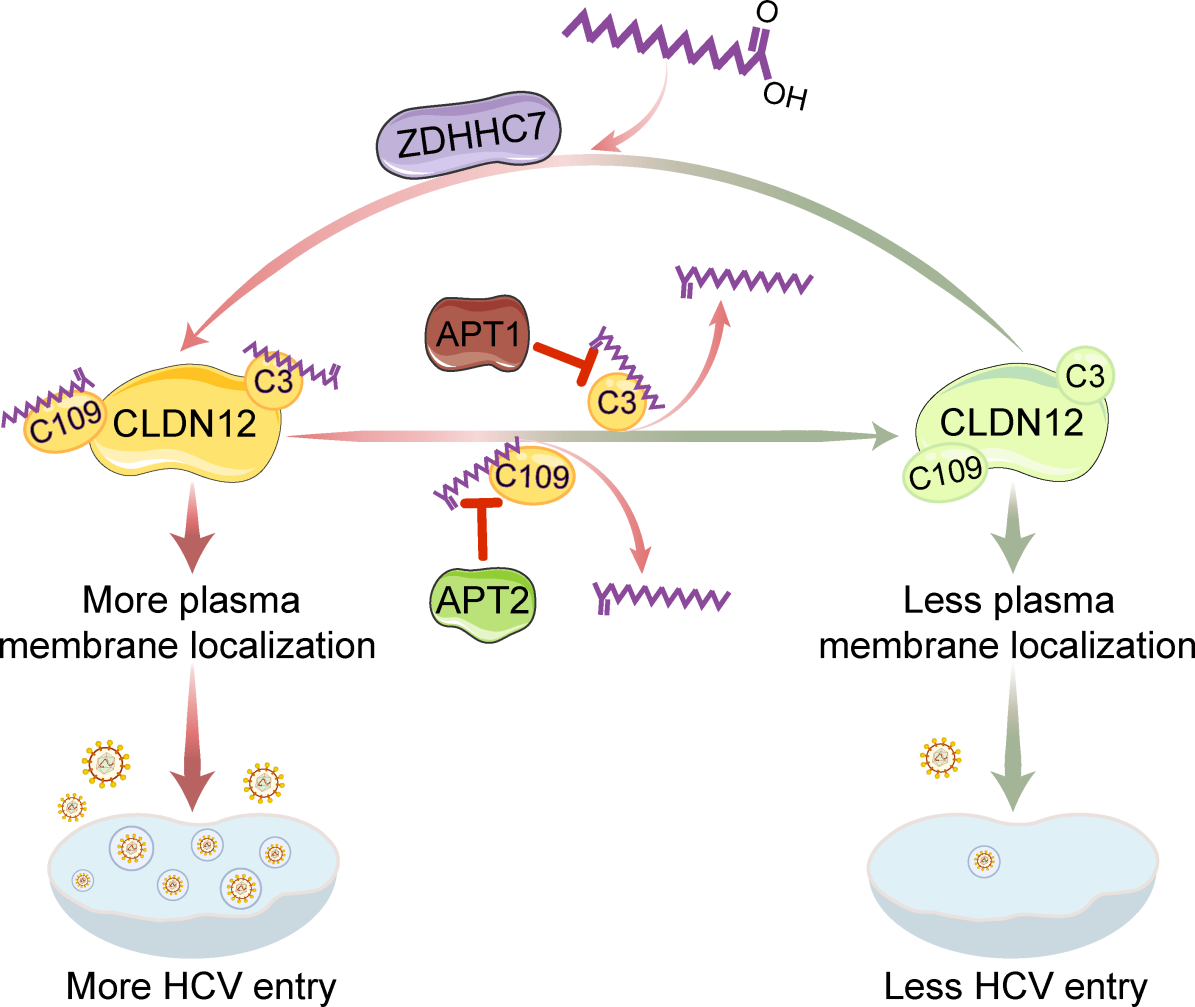
Model illustrating the key role of palmitoylation in CLDN12 during HCV entry.

In this study, we elucidated the dynamic palmitoylation switch regulating the function of the HCV entry co-receptor CLDN12 and its underlying mechanisms. The palmitoylation modification provides a reversible membrane anchor for CLDN12, dynamically regulating its functional microdomain distribution on the plasma membrane, thereby establishing an efficient and adjustable cellular entry portal for the virus. This significantly affects the HCV entry efficiency mediated by CLDN12, revealing a key molecular strategy by which the virus hijacks and utilizes the host protein post-translational modification system to enhance its infectious efficiency (Fig. 8).

We identified the third and 109th cysteine residues of the CLDN12 protein as functional palmitoylation modification sites. This modification regulates the plasma membrane localization level of CLDN12, thereby significantly enhancing HCV entry efficiency. Furthermore, we elucidated the dynamic regulation of this modification by the “PATs-APTs” regulatory enzymes: palmitoyl transferases such as ZDHHC7 catalyze the modification to promote viral entry, while acyl-protein thioesterases APT1 and APT2 play a negative regulatory role by specifically depalmitoylating the two sites. These findings not only provide potential synergistic therapeutic targets for future research but also collectively form a sophisticated and reversible “palmitoylation cycle” dynamic regulatory model, which fine-tunes the efficiency of CLDN12-mediated viral entry and offers new insights for designing strategies to modulate host cell susceptibility to viruses. This puts us in a position to potentially generate a synergistic effect with existing DAA therapies, particularly providing key complementary strategies for preventing reinfection, ultimately aiming to establish a more comprehensive antiviral treatment system.

The efficiency of CLDN12-mediated HCV entry is likely linked to the dynamic regulation of palmitoylation, which modulates the plasma membrane localization of CLDN12. Palmitoylation provides a hydrophobic anchor for membrane proteins, forming the basis for their functional expression (29). This process connects palmitic acid chains covalently to the cysteine residues at positions C3 and C109 of CLDN12, facilitating the embedding of these hydrophobic chains into the lipid bilayer of the cellular membrane. Such modifications enhance the hydrophobicity of CLDN12 and stabilize its membrane anchoring, preventing the protein from dissociating from the membrane or mislocalizing. Palmitoylation plays a crucial regulatory role in protein targeting and transport. Studies have demonstrated that the palmitoylation of CD36 targets it to the plasma membrane, wherein ZDHHC4 modifies newly synthesized CD36 for sorting to the membrane, while ZDHHC5 maintains its localization (30). For CLDN12, the final plasma membrane localization after modification is also tightly regulated, with palmitoyl transferases such as ZDHHC7 facilitating its transport from the site of synthesis to the plasma membrane and promoting its enrichment in functional membrane microdomains, such as lipid rafts. In summary, palmitoylation is a key regulator of CLDN12’s plasma membrane localization. Given the dynamic reversibility of this modification, the balance between “palmitoylation” and “depalmitoylation” directly determines the functional localization level of CLDN12 on the plasma membrane, which ultimately influences the surface density of functional HCV co-receptors and thus regulates viral entry efficiency.

The molecular mechanism by which CLDN12 palmitoylation facilitates HCV entry likely involves either enhancing the direct binding to the viral envelope glycoproteins (E1/E2) or aiding in downstream internalization processes. However, current evidence does not support direct binding between CLDN12 and HCV E1/E2 glycoproteins. Systematic screening studies indicate that CLDN12 cannot independently mediate HCV entry, and no research has shown a direct interaction between CLDN12 and E1/E2. Therefore, it is more plausible that CLDN12 palmitoylation exerts its effects through an indirect mechanism. Previous research has established that CLDN12 directly interacts with CD81, the primary receptor for HCV (18). Studies have also demonstrated that palmitoylation of CD81 regulates its interactions with other tetraspanins, such as CD151, thereby influencing the efficiency of HCV entry (25). Building on these findings, we propose a mechanistic model in which CLDN12 palmitoylation may impact the dynamic assembly or stability of the complex by modulating the interaction between CLDN12 and CD81, thus indirectly affecting HCV entry. Moreover, HCV particles can induce the internalization of CD81 and CLDN1, promoting co-endocytosis of the receptor complex and subsequent internalization of viral particles (31). Therefore, CLDN12 palmitoylation may play a role in regulating the internalization efficiency of the CLDN12-CD81 complex. The results of this study provide a foundation for future investigations into the specific molecular mechanisms through which CLDN12 palmitoylation influences HCV entry.

In the model we propose, CLDN12 palmitoylation plays a critical role in regulating HCV entry by influencing the assembly of the CLDN12-CD81 receptor complex and potentially affecting its internalization efficiency. To clarify which steps are supported by direct evidence and which are speculative, we outline each aspect in sequence. First, our data clearly show that mutations at palmitoylation sites or treatment with the APT2 inhibitor ML349 can alter CLDN12 palmitoylation levels. Second, co-immunoprecipitation experiments indicate that palmitoylation negatively impacts CLDN12-CD81 binding: reduced palmitoylation strengthens their interaction, while increased palmitoylation weakens it. Third, assays using HCVpp and HCVcc demonstrate that palmitoylation positively regulates HCV entry: decreased palmitoylation diminishes entry, while increased palmitoylation enhances it. However, we currently lack direct experimental support for the “internalization efficiency” aspect, as we have not measured the internalization of the CLDN12-CD81 complex. Concerning whether stronger complex assembly directly leads to more efficient internalization and subsequent viral entry, our findings suggest otherwise. The C3S/C109S mutant promotes complex assembly but hinders viral entry, whereas ML349 treatment reduces complex assembly yet increases viral entry. This indicates that the efficiency of complex assembly does not straightforwardly predict successful viral entry, and the actual changes in internalization efficiency remain uncertain. One possible explanation is that CLDN12 palmitoylation may influence both complex assembly and subsequent internalization pathways. With lower palmitoylation levels, enhanced assembly could redirect internalization toward non-productive routes, such as lysosomal degradation. Conversely, at higher palmitoylation levels, internalization might be directed to functional endosomal compartments that facilitate HCV fusion. Alternatively, internalization efficiency itself may be regulated independently by palmitoylation. Another hypothesis to explore is that palmitoylation, as a membrane-anchoring modification, might directly recruit endocytic adaptor proteins, regardless of complex assembly status. While the C3S/C109S mutations could cause local conformational changes, this does not alter our main conclusion that the palmitoylation status of CLDN12 regulates HCV entry. Differentiating between these mechanisms will be an important focus for future research. Ultimately, our data highlight that CLDN12 palmitoylation is a dynamic regulator of HCV entry and that the interplay between receptor complex assembly and effective viral entry is far more intricate than previously recognized.

As a cargo protein of COPII vesicles, CLDN12 is transported from the endoplasmic reticulum to the Golgi apparatus via COPII, and then further to the plasma membrane (18). Based on experimental results, we hypothesize that the key palmitoyl transferase ZDHHC7, located in the Golgi apparatus, first undergoes self-palmitoylation, which subsequently catalyzes the palmitoylation modification of specific cysteine residues on the substrate CLDN12. Following this modification, CLDN12 is packaged into transport vesicles directed toward the plasma membrane, where it anchors and executes its role in mediating viral entry.

Our study found that both APT1 and APT2 act on the two palmitoylation sites of CLDN12 but exhibit a degree of site preference, likely due to their different subcellular localizations and the accessibility of substrate sites. The depalmitoyl transferase APT1 is distributed across various cellular compartments, including the plasma membrane, cytoplasm, endoplasmic reticulum, and mitochondria, while APT2 primarily resides in the cytoplasm (32–34). Both are involved in palmitoylation modification within the Golgi apparatus, after which APT1 facilitates depalmitoylation and mediates the depalmitoylation process of APT2, collectively executing the function of regulating substrate depalmitoylation (34, 35). CLDN12 is a protein with four transmembrane domains (TMD1–TMD4), including a shorter cytoplasmic N-terminus, an extended cytoplasmic C-terminus, two extracellular domains, and a short intracellular loop. The N-terminal peptide interacts synergistically with the adjacent TMD1, which is crucial for the correct insertion, folding, and early stability of the protein in the endoplasmic reticulum (36). The intracellular loop of CLDN12 typically stays close to the inner surface of the plasma membrane or interacts with membrane lipids and other transmembrane domains; thus, its conformation is restricted. The C3 site is located in the N-terminal peptide of CLDN12, while the C109 site is located in its intracellular loop. APT1 performs its depalmitoylation action in the cytoplasm, which more easily targets accessible substrates exposed to the cytosol, primarily regulating soluble proteins and peripheral membrane proteins (e.g., G protein alpha subunits and non-receptor tyrosine kinases like Src family) (32, 37). Given that the C3 site is fully exposed in the cytoplasm and its surrounding amino acid side chains are well extended, we speculate that APT1 favors acting on this site to regulate the rapid, dynamic cycling of CLDN12 between the plasma membrane and endosomes: namely, when APT1 activity is elevated, the recycling of CLDN12 from the plasma membrane accelerates. APT2 predominantly exists in the cytoplasm in a free form, requiring recruitment to the membrane through electrostatic attraction, hydrophobic loop insertion, and PAT-mediated S-acylation steps. APT2 is a key depalmitoylation enzyme regulating integral membrane proteins, particularly four-transmembrane proteins such as CD9 and CD81, which insert with a “β-hairpin” structure and bend the lipid bilayer to extract the substrate acyl chain. This unique membrane action mechanism makes APT2 more effective than APT1 for palmitoylation sites embedded deep in the membrane environment of integral membrane proteins (38). Therefore, APT2 is more inclined to regulate the C109 site located in the short intracellular loop. In summary, the differential regulation of the two sites by APT1 and APT2 allows cells to independently and cooperatively control the transition of the same protein from a basic membrane-anchored state to a dynamically regulated state for viral binding and internalization.

Members of the claudin protein family exhibit a conserved four-transmembrane topology (39, 40). This structural characteristic implies that the cysteine residues on their intracellular domains (such as the N-terminus, intracellular loops, and C-terminus) are potential targets for lipid modifications such as palmitoylation, which has been confirmed to be a core mechanism regulating the dynamic localization and function of family proteins (41, 42). For instance, the expression of CLDN3 correlates positively with ovarian cancer pathology, and S-palmitoylation mediated by ZDHHC12 affects the protein’s membrane localization and tight junction function, making targeting this modification process a potential strategy for treating malignant ovarian cancer (43). In liver cancer, the palmitoylation of residues C104 and C107 of CLDN4 enhances its membrane stability and inhibits ubiquitin-mediated degradation, thus driving lineage transition and drug resistance in cancer cells (44). Insulin maintains lymphatic endothelial barrier function through the palmitoylation regulatory mechanism of CLDN5, revealing its important interactions with insulin signaling and lipid metabolism (45). CLDN6 indirectly inhibits palmitoylation of downstream RAS proteins by suppressing SREBP1, thereby inhibiting breast cancer progression, suggesting that monitoring CLDN6 expression and targeting this palmitoylation pathway is a potential therapeutic strategy for RAS-driven breast cancer (46). The palmitoylation modification of CLDN7 is crucial for its localization within specific membrane domains and its interactions with proteins such as EpCAM, as this modification influences whether CLDN7 participates in tight junction formation or drives cell migration and invasion, thereby regulating its tumor suppressor or cancer-promoting functions in cancer (47). Collectively, these studies demonstrate that palmitoylation plays a key role in the localization and functional regulation of claudin proteins, a mechanism considered a broad consensus for this protein family. Therefore, the relevant findings of this study regarding CLDN12’s palmitoylation not only substantiate this perspective from structural biology and family commonality but also extend our understanding of this post-translational modification’s regulatory role in transmembrane proteins, particularly viral host factors.

It is already known that HCV viral protein NS2 undergoes palmitoylation, which can simultaneously regulate both viral RNA replication and virus particle assembly (24). The palmitoylation of the viral receptor CD81 is vital for the effective invasion of cells by HCV through this receptor (25, 48). This study reveals the mechanism by which CLDN12 promotes HCV entry through palmitoylation, expanding the range of palmitoylation substrates related to HCV host proteins. HCV infection profoundly alters the metabolic pathways of host liver cells, with one of its core strategies being the induction of *de novo* synthesis of the host’s fatty acids, providing a critical metabolic foundation for establishing an efficient self-sustaining infection loop. Early studies showed that HCV infection significantly upregulates the expression and activity of fatty acid synthase, thereby increasing the biosynthesis of endogenous palmitic acid in liver cells (21–23). The palmitic acid produced by FASN is further converted to palmitoyl-CoA, serving as a direct donor for the palmitoyl transferases of the ZDHHC family, catalyzing the covalent attachment of palmitoyl groups to cysteine residues of substrate proteins such as CLDN12. The increased palmitoylation level of CLDN12 enhances its plasma membrane localization, thus optimizing its mediation of HCV entry efficiency. As a result, the virus successfully establishes a self-reinforcing positive feedback loop: the infection itself creates metabolic conditions that enhance subsequent entry capabilities, thereby promoting the establishment and persistence of the infection.

From a druggability perspective, the regulatory enzymes of S-palmitoylation are highly promising drug targets. On the one hand, the conserved DHHC zinc finger domain of the palmitoyl transferase family provides a clear target for designing small-molecule inhibitors (49). For example, targeting ZDHHC7 (e.g., through gene knockout or using its specific inhibitor MY-D4) has shown clear efficacy in animal models of inflammatory bowel disease (50, 51). On the other hand, acyl-protein thioesterases, as serine hydrolases, also have active sites suitable for the development of small-molecule agonists. Existing studies have validated that their enzymatic activity can be precisely regulated by small molecules through the development of inhibitors (52). These advances collectively confirm the feasibility of developing small-molecule drugs targeting PATs and APTs, marking a transition in this field from basic research to translational medicine. Based on this, the core translational significance of this study lies in establishing the key enzymes regulating the dynamic balance of CLDN12 palmitoylation—palmitoyl transferase ZDHHC7 and the depalmitoylating enzymes APT1/2—as potential novel host targets for the prevention and treatment of HCV infection. Accordingly, we propose two targeting strategies: one as a prevention strategy aiming to develop ZDHHC7 inhibitors or APT1/2 agonists to reduce the level of functional CLDN12 on the surface of hepatocytes, thereby establishing an immune barrier against the initial HCV infection for high-risk populations such as liver transplant recipients; the second is a combination therapy strategy, intending to use the aforementioned modulators in conjunction with existing DAAs to achieve more thorough viral clearance, treat reinfections, and potentially delay or reduce the risk of resistance. Although translating the CLDN12 palmitoylation regulatory network into clinical therapies faces numerous challenges, advances in related fields provide robust evidence for this strategy. For example, antibodies targeting other tight junction proteins (such as CLDN1) have shown potential in clinical trials aimed at preventing HCV reinfection after liver transplantation (53). Additionally, exploring targeting palmitoylation pathways in cancer, inflammation, and other diseases is an emerging focus. These factors lay a solid theoretical foundation for HCV prevention and treatment strategies based on ZDHHC7 and APT1/2 while showcasing clear translational prospects.

Despite the new strategies this study offers for targeting CLDN12 palmitoylation to prevent and treat HCV infection, some significant limitations remain. First, the core molecular mechanisms were primarily validated in cell line models and need further confirmation in more complex models that closely mimic human physiological environments, such as primary human liver cells or humanized mouse models. Second, the predicted membrane topology that guided the selection of the candidate palmitoylation sites C3 and C109 of CLDN12 is currently based solely on the UniProt computational model and has not been experimentally validated. Future studies employing biochemical topology approaches, such as glycosylation mapping or protease accessibility analysis, are necessary to confirm the actual cytoplasmic accessibility of these residues. Third, the expression levels and dynamic enzymatic activity of the key regulatory enzymes ZDHHC7 and APT1/2 in liver tissues of chronic HCV-infected patients are still unclear, necessitating future clinical sample analyses to explore their correlations with disease progression, thereby strengthening the clinical translational rationale for this strategy. To facilitate clinical translation, future studies could explore several directions. First, researchers should conduct high-throughput drug screening that targets the DHHC domain of ZDHHC7 or the active sites of APT1/2. This approach aims to identify lead compounds, whether inhibitors or agonists, that exhibit high activity and selectivity. Second, it is important to broaden the potential applications of this target by investigating whether the dynamic regulation of CLDN12 palmitoylation influences its other functions. Third, in-depth research should be performed on the interactions between the virus and the host. Specifically, this research could explore whether HCV actively modulates the palmitoylation switch by upregulating ZDHHC7 or inhibiting APT1/2 during the infection process, either through its viral proteins or by hijacking host signaling pathways to enhance its own invasion efficiency. Those future researches will not only deepen our understanding of the pathogenesis of HCV but may also reveal potential new mechanisms of viral escape.

It is important to note that the role of CLDN12 in HCV entry identified in this study is partial and non-essential. CLDN12 functions more as a regulatory or auxiliary factor rather than being crucial like CD81 or CLDN1. Therefore, the primary contribution of this study is not in quantifying how much CLDN12 influences viral infection efficiency, but in uncovering a novel regulatory mechanism for an auxiliary entry factor—reversible palmitoylation. Our findings show that the palmitoylation status of CLDN12 dynamically regulates its auxiliary function during HCV entry, which is a regulatory aspect not previously studied in-depth. This discovery enhances our understanding of post-translational modifications in tight junction proteins and suggests that other auxiliary factors, and even essential receptors, might also be regulated by similar reversible palmitoylation. From a therapeutic standpoint, targeting a regulatory host factor such as CLDN12 may provide a better therapeutic index and increased safety compared to targeting essential entry receptors, a concept that deserves further exploration.

In summary, the tight junction protein CLDN12 undergoes dynamic palmitoylation modifications at cysteine residues C3 and C109, primarily catalyzed by the palmitoyl transferase ZDHHC7, promoting the HCV entry process mediated by CLDN12. In contrast, acyl-protein thioesterases APT1 and APT2 are responsible for removing palmitoyl groups from CLDN12, with differential effects between sites, and negatively regulate HCV entry through depalmitoylation. These results reveal that ZDHHC7 and APT1/2 compose a finely tuned reversible network of post-translational modification regulation, dynamically controlling the function and localization of host factors during HCV infection. Therefore, the palmitoylation level of CLDN12 and its regulatory enzymes ZDHHC7, APT1, and APT2 are key factors influencing HCV entry efficiency. This discovery not only elucidates a key molecular strategy by which HCV optimizes its infectious efficiency by hijacking the host protein post-translational modification system, but also provides a new theoretical basis for understanding virus-host interactions and for developing antiviral therapies targeting palmitoylation.

## MATERIALS AND METHODS

### Cells and reagents

Huh7.5.1 (RRID:CVCL-E049), obtained from Dr. Francis Chisari, HeLa (RRID:CVCL_0030), and HEK-293T (RRID:CVCL_0063) were maintained in Dulbecco’s modified Eagle’s medium (DMEM, KeyGen BioTECH KGL1211-500, China) supplemented with 10% fetal bovine serum (FBS, ExCell, FSP500) and 1% penicillin-streptomycin 100 × solution (HyClone, SV30010, USA). All cell lines were maintained at 37°C in a 5% CO_2_ atmosphere and were routinely tested for mycoplasma contamination. DMSO (D8371) was obtained from Solarbio, China. FITC-labeled wheat germ agglutinin (WGA) (GTX01502, RRID:AB_1657336) was obtained from GeneTex, USA. ML348 (HY-100736) and ML349 (HY-100737) were obtained from MedChemExpress, USA. 2-BP (21,604) was obtained from Sigma, USA.

### Plasmids

The laboratory constructed expression plasmids for mCherry-tagged CLDN12 (mCherry-CLDN12) and its mutants (C3S/C109S). The specific process is as follows: first, the coding sequence of human CLDN12 was obtained and cloned into the mammalian expression vector pcDNA3.1(+) with an mCherry C-terminal tag. The C3S and C109S mutants were constructed by AZENTA Biotechnology Co., Ltd. (Suzhou, China) using site-directed mutagenesis to replace the cysteine residues at positions 3 and 109 of the mCherry-CLDN12 plasmid with serine. Ultimately, both the wild-type and mutant CLDN12 genes were integrated into the pcDNA3.1(+) vector for subsequent mammalian cell expression experiments. Additionally, plasmids expressing Flag-tagged ZDHHC family members (ZDHHC1 to ZDHHC24) were obtained from Sino Biological (Beijing, China) (54). Plasmids expressing Flag-tagged APT1 and APT2 were purchased from Sino Biological. All ZDHHC and APT plasmids carried a CMV promoter and a kanamycin resistance gene. The plasmids encoding APT1-S119A, APT2-S122A, and ZDHHC7 C160S-Flag were constructed by AZENTA Biotechnology Co., Ltd (Suzhou, China). Jc1Flag(p7-nsGluc2A) was obtained from Dr. Charles Rice (55). Expression plasmids encoding HIV Gag/Pol (pLP1), HIV Rev (pLP2), pLenti6 encoding luciferase, and HCV E1E2 from genotype 1b strain Con1 were provided by Dr. Ping Zhao (56, 57).

### HCV Jc1 infection

The HCV Jc1 virus used in this study was generated through plasmid linearization from Jc1Flag(p7-nsGluc2A) and *in vitro* transcription, and subsequently packaged by electroporation into Huh7.5.1 cells. The viral yield was quantitatively monitored using its *Gaussia* luciferase reporter system. The resulting virus was assessed using the Gaussia-Lumi *Gaussia* luciferase reporter gene detection kit (RG072M, Beyotime, China), with readings taken from the supernatant after cell debris was removed. Supernatants with high readings (greater than 10^7^ RLU) were preserved and served as seed virus for subsequent infection experiments. Huh7.5.1 cells were plated at a density of 70%–80%, and then the medium was replaced with DMEM containing 2% fetal bovine serum, followed by the addition of the appropriate amount of seed virus. The inoculum of the virus was adjusted based on the *Gaussia* luciferase readings of the seed virus: if the reading was below 1 × 10^7^, the virus amount was increased, and if it was above 1 × 10^8^, the amount was decreased. The cell growth status and density were continuously monitored, and when the cell density exceeded 95%, passaging was performed at a ratio of 1:4. The above viral infection steps were repeated until the cells displayed significant cytopathic effects, and the density could not be restored to above 90%. At this point, the supernatant was collected, and the *Gaussia* luciferase activity was measured again, preserving the virus.

In this study, Huh7.5.1 cells were seeded in a 6-well plate at a density of 2 × 10^5^ cells per well and cultured at 37°C with 5% CO_2_ until they reached 60%–80% confluence. A pre-stored viral solution with a *Gaussia* luciferase reading higher than 1 × 10^8^ RLU was selected, and 6–8 different concentrations of viral dilutions were prepared using a 2-fold serial dilution method. Cells were infected with these dilutions, with at least two technical replicates for each concentration. After 48 h, the cell supernatant was collected. In a 96-well plate, 30 μL of each diluted supernatant sample was added sequentially, followed by an equal volume of *Gaussia* luciferase substrate working solution, with three technical replicates for each sample. After equilibrating at room temperature for 10 min, the chemiluminescence signal was detected using the Synergy Neo2 multifunctional microplate reader. The optimal dilution concentration for subsequent HCV JC1 viral infection experiments was determined as the lowest dilution factor at which the luminescence signal approached the platform value of the original viral solution, and cell infection was nearly saturated.

For functional experiments, Huh7.5.1 cells were seeded in a 24-well plate at a density of 5 × 10^4^ cells per well, and once they reached 60%–80% confluence, the medium was changed to DMEM containing 2% fetal bovine serum. The transfection reagent FuGENE was used to transfect plasmids expressing the target protein, with a blank vector or DMSO as a control. After 6 h of transfection, the medium was replaced with complete culture medium or treated with the corresponding drugs, followed by infection of the cells with the virus at the optimal dilution concentration. After continuing to culture for 6 h, the viral solution was removed, and the cells were washed three times with PBS and then replaced with complete DMEM containing 10% fetal bovine serum for further culture. After 18 h, the supernatant was collected for chemiluminescence detection, and cell viability was assessed using the CCK-8 method, with *Gaussia* luciferase signals normalized to cell viability to enhance data accuracy. Gene knockdown experiments followed the same infection and detection protocols, with the cells pre-treated with the corresponding siRNA.

### Package of HCVpp and VSVpp

The preparation of HCVpp and VSVpp is achieved through co-transfection of HEK-293T cells. The transfection system included the following plasmids: the pLenti6 vector encoding a luciferase reporter gene, a plasmid expressing HIV Gag/Pol (pLP1), a plasmid expressing HIV Rev (pLP2), and a plasmid encoding the HCV E1E2 glycoproteins (for HCVpp) or VSVG. After 72 h of transfection, the cell supernatant containing HCVpp is collected and used to infect HEK-293T cells. After 48 h of infection, the cells are collected and lysed using lysis buffer. The lysate is centrifuged at 13,680 × *g* for 30 min at 4°C, and the supernatant is collected. Subsequently, the luciferase activity in the supernatant was measured using the Luciferase Assay System Freezer Pack (Promega, E4530) to evaluate the infection efficiency of HCVpp.

### Antibodies

The antibodies utilized in this study included rabbit anti-Flag (XHY021L, Xuheyuan, Beijing, China), rabbit anti-mCherry (XHY027L, Xuheyuan, Beijing, China), rabbit anti-Na^+^/K^+^-ATPase (14418-1-AP, Proteintech, Chicago, USA), mouse anti-Flag (XHY001L, Xuheyuan, Beijing, China), mouse anti-mCherry (XHY039L, Xuheyuan, Beijing, China), mouse anti-α-tubulin (66031-1-Ig, Proteintech, Chicago, USA), and mouse IgG (H + L) (bs-0296P, Bioss, Beijing, China). Western blot detection was performed using HRP-conjugated goat anti-mouse IgG (RRID:AB_856214, SA00001-1, Proteintech, China) and HRP-conjugated goat anti-rabbit IgG (RRID:AB_10890902, SA00001-2, Proteintech, China). Alexa Fluor 405-conjugated goat anti-mouse secondary antibody (AB_2536180) was obtained from Thermo Fisher Scientific Inc. (Waltham, MA, USA).

### Prediction of palmitoylation sites

Potential palmitoylation sites in the human protein CLDN12_HUMAN (UniProtKB ID: P56749) were predicted using the CSS-Palm 4.0 software. The prediction threshold was set to “medium,” while all other parameters were set to the default values of the software. The UniProtKB ID was then imported into SnapGene software for subcellular localization analysis of the two predicted potential palmitoylation sites (both cysteine residues), confirming that both are located on the cytoplasmic side of the protein. The results of this localization analysis provided the necessary bioinformatics basis for subsequent targeted functional validation experiments of palmitoylation.

### Co-immunoprecipitation

Transfection was conducted when the cells in a 6-well plate reached 70%–80% density. Each well was transfected with 1 μg of plasmid DNA, which was mixed with polyethyleneimine (PEI) at a weight-to-volume ratio of 1:2 (μg:μL). The mixture was incubated at room temperature for 15 min to form DNA-PEI complexes and then added to high-glucose DMEM for transfection. After 24 h, cells were collected for further processing. For each sample, 20 μL of Protein A + G agarose beads (P2055, Beyotime, Beijing, China) was used. The beads were washed three times with pre-chilled phosphate-buffered saline (PBS) (P1010, Solarbio, Beijing, China) to equilibrate them. After equilibrating, the beads were co-incubated with the corresponding antibody at 4°C for 1 h to facilitate binding. The cells were washed three times with pre-chilled PBS before being lysed using a pre-chilled lysis buffer (50 mM Tris-HCl pH 7.4, 300 mM NaCl, 1% Triton X-100), supplemented with a 1 × protease inhibitor mixture for mammalian cells and tissues (P1010, Beyotime, Beijing, China). The lysate was centrifuged at 12,000 rpm (13,680 × *g*, HERMLE Z216MK centrifuge) at 4°C for 15 min, and the supernatant was collected. The supernatant was incubated with the Protein A + G agarose beads bound to antibodies at 4°C for 1.5 h. The beads were then washed three times with a wash buffer (50 mM Tris-HCl, pH 7.4, 300 mM NaCl, 0.1% Triton X-100) to remove non-specific binding proteins and impurities.

### Plasma membrane enrichment experiment

The kit (BB-31161) for plasma membrane enrichment experiment is from BestBio (Shanghai, China). Cells (5 × 10⁶ to 1 × 10⁷) were collected and centrifuged at 4°C for 5 min at 500 × *g*. The supernatant was discarded, and the cells were washed twice with cold PBS. To 500 μL of cold Extraction Buffer A, 5 μL of Reagent B and 2 μL of the protease inhibitor mixture are added, then mixed well and kept on ice. Then, 300–500 μL of the above premixed solution was added to the cell pellet, and the cells were resuspended by high-speed vortexing. The suspension was incubated with constant shaking at 2–8°C for 30 min. It was then vortexed again for 5 s and centrifuged at 4°C for 5 min at 12,000 × *g*. The supernatant was transferred to a new tube and incubated in a 37°C water bath for 5–10 min, followed by centrifugation at 37°C for 3 min at 500–1,000 × *g*. At this point, the solution separates into two layers. The lower layer (30–50 μL) is the plasma membrane proteins. The upper layer was carefully removed, and the lower layer proteins were dissolved in 50–150 μL of cold Reagent C with thorough mixing. The resulting plasma membrane proteins can be aliquoted and stored at −80°C or used directly for downstream experiments.

### Western blot

After separation by SDS-PAGE, the protein samples were transferred to a methanol-preactivated PVDF membrane. For the co-immunoprecipitation experiments, the PVDF membrane was blocked in TBST buffer (pH 8.0; CWBIO, CW0043S) containing 5% non-fat dry milk, followed by incubation with the corresponding primary and secondary antibodies. In the acyl-biotin exchange assay, blocking of the PVDF membrane was performed with TBST buffer containing 3% bovine serum albumin (BSA; ST023, Beyotime, Beijing, China). Finally, all protein signals were detected and imaged using enhanced chemiluminescence.

### ABE assay

To determine the palmitoylation level of mCherry-CLDN12, this study employed the acyl-biotin exchange assay. The composition of the cell lysis buffer was as follows: 50 mM Tris-HCl (pH 7.5) (T1140, Solarbio, Beijing, China), 150 mM NaCl, 10% glycerol, 1% IGEPAL CA-630 (I3021, Sigma-Aldrich, St. Louis, MO, USA), supplemented with a protease inhibitor mixture (100×, Beyotime, P1010), 0.1 mM PMSF (ST506, Beyotime, Beijing, China), and 50 mM N-ethylmaleimide (NEM) (E100553, Aladdin, Shanghai, China). After lysis, NEM was used to pre-block free thiol groups in the proteins, followed by immunoprecipitation. The precipitated complexes were treated with hydroxylamine (HAM) (H828371, MACKLIN, Shanghai, Beijing) to expose palmitoylated cysteine residues and subsequently labeled with biotin-BMCC (B2112, ProteoChem, Shanghai, Beijing). Finally, biotin labeling signals were detected using streptavidin-HRP (M00091, GenScript, Nanjing, Jiangsu, China) through Western blotting, reflecting the palmitoylation level of CLDN12.

### siRNA transfection

The siRNA used in this study was custom-synthesized by GenePharma. Cell transfection was performed using Lipofectamine RNAiMAX (13,778,150, Invitrogen, Carlsbad, CA, USA) according to the manufacturer’s recommended protocol, at a ratio of 1 μL of siRNA (10 μM) per 10^5^ cells, with a transfection duration of 48 h. The sequences of siRNA are listed below: CCAAGGAUCUUCCCAUCUAUU (ZDHHC2 siRNA), UUUCGUAGCGUUUCCUUUGGG (ZDHHC7 siRNA), CCUUCCCUAUGAGGUCUAUTT (ZDHHC15 siRNA), GCAAGAGCUUUACCUAUCU (ZDHHC20 siRNA), AGAUAGGUAAAGCUCUUGC (ZDHHC20 siRNA), CCUCGCUGGGAACGCACUUTT (CLDN12 siRNA), and UUCUCCGAACGUGUCACGUTT (negative control siRNA).

### Quantitative real-time PCR

Total RNA was extracted using the FastPure Cell/Tissue Total RNA Isolation Kit V2 (RC112-01, NuoViva, Nanjing, Jiangsu, China). Subsequently, according to the manufacturer’s instructions, 1 μg of RNA was reverse-transcribed into cDNA using the PrimeScript Reverse Transcriptase Kit (RR047A, Takara, Kyoto, Japan). Gene expression quantification was performed using real-time quantitative PCR (RT-qPCR), with the reaction system containing TB Green Fast qPCR Mix (RR430A, Takara, Japan) and specific primers, using GAPDH as the reference gene. All experiments were independently repeated three times, and the relative gene expression levels were calculated using the 2-ΔΔCt method. The primers are listed below: ZDHHC2 F: 5′-GAAAACACTGGCGAACAAGT-3′,

ZDHHC2 R: 5′-GCCAGTGGCTCCATTTCTTT-3′; ZDHHC7 F: 5′-TGCTCACCGACCCTGAAAAA-3′, ZDHHC7 R: 5′-CACCACAAATGCCCACAAGG-3′; ZDHHC15 F: 5′-TATTGGTTCCAGCCCTGGTG-3′, ZDHHC15 R: 5′-TGCTAGCAGTGGGTTCTGTG-3′; ZDHHC20 F: 5′-GCTCAGCCTGTGACTCATGT-3′, ZDHHC20 R: 5′-CTGTTGCAGCCACGAAAAGG-3′; CLDN12 F: 5′-CAGTTTGCCCTACCCCTCAG-3′, CLDN12 R: 5′-CAGTTTGATGTTGGGCACCG-3′; GAPDH F: 5′-GGAGCGAGATCCCTCCAAAAT-3′, GAPDH R: 5′-GCTGTTGTCATACTTCTCATGG-3′.

### Immunofluorescence microscopy

Cells grown on cover slips were gently washed with PBS and fixed with pre-chilled methanol at 4°C for 15 min. After fixation, the cells were blocked in PBS containing 10% goat serum at room temperature for 10 min to reduce non-specific binding. Following blocking, the cover slips were incubated with the primary antibody diluted 1:200 at room temperature for 1 h. After removing the primary antibody, the cells were washed three times with blocking solution and then incubated with the goat anti-mouse Alexa Fluor 405-labeled secondary antibody, diluted 1:200, at room temperature in the dark for 1 h. To label the cell membrane, the samples were further stained with WGA conjugated to a green fluorophore. After staining, the samples were mounted with an anti-fade mounting medium to maintain fluorescence stability. All samples were observed using an Olympus FV12-IXCOV dual-view confocal microscope (Japan), employing a 100× oil immersion objective to collect images with a resolution of 1,024 × 1,024 pixels. At least 10 independent fields were randomly captured for each slide, and subsequent image analysis was performed using ImageJ software (RRID:SCR_003070).

The following is a detailed description of the methods used for image acquisition and quantitative fluorescence signal analysis. First, it is crucial that the original images used in our analysis maintain consistent acquisition parameters (such as laser intensity, gain, exposure time) across different treatment groups within the same experimental set. During the statistical analysis, we utilized ImageJ to open the merged images. We cropped the areas where colors from different channels overlapped—for example, where red and green merge to create yellow or where red, green, and blue combine to produce white. For RGB images, we separated them into individual channels and applied appropriate pseudocolors, adjusting the thresholds for both 16-bit and 8-bit images. To reduce the potential for errors from manual adjustments, we relied on the default threshold settings in the software (marked as “Red”) and selected the “Dark Background” option. We ensured that the threshold settings remained consistent for both the mock and experimental groups within the same series of experiments. After isolating the relevant areas, we selected the measurement parameters “Mean gray value” and “Limit to threshold.” Once we identified the desired regions, we clicked “Measure” to obtain the corresponding values. Finally, we normalized the statistical data for both the mock and experimental groups. The quantitative results were primarily presented in the form of statistical graphs to ensure an objective representation of the data differences.

### CCK-8 assay

Huh7.5.1 cells were seeded in a 24-well plate (with 500 μL of complete DMEM containing 10% fetal bovine serum and 1% penicillin-streptomycin solution per well, at a density of 2 × 10^4^ cells) and cultured for 3 days. Four hours before the end of the incubation period, a complete medium containing the CCK-8 reagent (PF00004, Proteintech, USA) was prepared according to the manufacturer’s instructions (CCK-8 volume: complete medium volume = 1:30) and added to each well (300 μL). The absorbance at 450 nm for each well was measured using a multifunctional microplate reader (Synergy Neo2).

### Cell viability assays

The assessment of cell viability was based on the measurement of intracellular ATP levels. Specifically, the CellTiter-Glo Luminescent Cell Viability Assay kit (C0065S, Beyotime, Beijing, China) was used for this detection, and the experimental procedures were conducted exactly according to the manufacturer’s provided manual.

### Statistics

ImageJ software was utilized for statistical analysis of fluorescence intensity. The statistical analyses were conducted using GraphPad Prism software version 10.1.2 (RRID:SCR_002798). The unpaired two-tailed multiple *t*-tests were used for comparisons between two groups, and one-way ANOVA was used for comparisons among three or more groups. All values are depicted as mean ± standard deviation. A *P* value of more than 0.05 was considered statistically not significant (ns). *, *P*  < 0.05; **, *P* < 0.01; ***, *P* <   0.001; ****, *P*  < 0.0001.

## Data Availability

The authors confirm that all data underlying the findings are fully available without restriction. All relevant data are within the paper.
